# Physical activity and sedentary behaviour interventions for people living with both frailty and multiple long-term conditions and their informal carers: a scoping review and stakeholder consultation

**DOI:** 10.1093/ageing/afae255

**Published:** 2024-11-19

**Authors:** Hannah M L Young, Joseph Henson, Paddy C Dempsey, Scott A Willis, Roseanne E Billany, Ffion Curtis, Laura Gray, Sharlene Greenwood, Louisa Y Herring, Patrick Highton, Ryan J Kelsey, Selina Lock, Daniel S March, Krishna Patel, Jack Sargeant, Harini Sathanapally, Avan A Sayer, Martha Thomas, Noemi Vadaszy, Emma Watson, Tom Yates, Melanie Davies

**Affiliations:** Leicester Diabetes Centre, College of Life Sciences, University of Leicester, Leicester, UK; Therapy Department, University of Hospitals of Leicester NHS Trust, Leicester, UK; NIHR Leicester Biomedical Research Centre, University of Leicester and University Hospitals of Leicester NHS Trust, Leicester, UK; Leicester Diabetes Centre, College of Life Sciences, University of Leicester, Leicester, UK; NIHR Leicester Biomedical Research Centre, University of Leicester and University Hospitals of Leicester NHS Trust, Leicester, UK; Leicester Diabetes Centre, College of Life Sciences, University of Leicester, Leicester, UK; MRC Epidemiology Unit, Institute of Metabolic Science, Cambridge University, Cambridge Biomedical Campus, Cambridge, UK; Baker Heart and Diabetes Institute, Physical activity and behavioural epidemiology laboratory, Melbourne, Australia; Institute for Physical Activity and Nutrition (IPAN), School of Exercise and Nutrition Sciences, Deakin University, Geelong, Victoria, Australia; NIHR Leicester Biomedical Research Centre, University of Leicester and University Hospitals of Leicester NHS Trust, Leicester, UK; National Centre for Sport and Exercise Medicine, School of Sport, Exercise and Health Sciences, Loughborough University, Loughborough, UK; NIHR Leicester Biomedical Research Centre, University of Leicester and University Hospitals of Leicester NHS Trust, Leicester, UK; Department of Cardiovascular Sciences, University of Leicester, Leicester, UK; Liverpool Reviews & Implementation Group (LRiG), University of Liverpool, Liverpool, UK; NIHR Leicester Biomedical Research Centre, University of Leicester and University Hospitals of Leicester NHS Trust, Leicester, UK; Department of Population Health Sciences, University of Leicester, Leicester, UK; Department of Renal Medicine, King’s College Hospital NHS Trust, London, UK; Renal Sciences, Faculty of Life Sciences and Medicine, King’s College London, London, UK; Leicester Diabetes Centre, College of Life Sciences, University of Leicester, Leicester, UK; NIHR Leicester Biomedical Research Centre, University of Leicester and University Hospitals of Leicester NHS Trust, Leicester, UK; Leicester Diabetes Centre, College of Life Sciences, University of Leicester, Leicester, UK; NIHR Applied Research Collaboration East Midlands, Leicester General Hospital, Leicester, UK; Leicester Diabetes Centre, College of Life Sciences, University of Leicester, Leicester, UK; NIHR Leicester Biomedical Research Centre, University of Leicester and University Hospitals of Leicester NHS Trust, Leicester, UK; Library Research Services, University of Leicester, Leicester, UK; NIHR Leicester Biomedical Research Centre, University of Leicester and University Hospitals of Leicester NHS Trust, Leicester, UK; Department of Cardiovascular Sciences, University of Leicester, Leicester, UK; Centre for Ethnic Health Research, University Hospitals of Leicester NHS Trust, Leicester, UK; Leicester Diabetes Centre, College of Life Sciences, University of Leicester, Leicester, UK; NIHR Leicester Biomedical Research Centre, University of Leicester and University Hospitals of Leicester NHS Trust, Leicester, UK; NIHR Applied Research Collaboration East Midlands, Leicester General Hospital, Leicester, UK; AGE Research Group, NIHR Newcastle Biomedical Research Centre, Translational and Clinical Research Institute, Faculty of Medical Sciences, Newcastle University and Newcastle Upon Tyne Hospitals NHS Foundation Trust, Newcastle-Upon-Tyne, UK; Leicester Diabetes Centre, College of Life Sciences, University of Leicester, Leicester, UK; NIHR Leicester Biomedical Research Centre, University of Leicester and University Hospitals of Leicester NHS Trust, Leicester, UK; Department of Population Health Sciences, University of Leicester, Leicester, UK; NIHR Leicester Biomedical Research Centre, University of Leicester and University Hospitals of Leicester NHS Trust, Leicester, UK; Department of Cardiovascular Sciences, University of Leicester, Leicester, UK; Leicester Diabetes Centre, College of Life Sciences, University of Leicester, Leicester, UK; NIHR Leicester Biomedical Research Centre, University of Leicester and University Hospitals of Leicester NHS Trust, Leicester, UK; Leicester Diabetes Centre, College of Life Sciences, University of Leicester, Leicester, UK; NIHR Leicester Biomedical Research Centre, University of Leicester and University Hospitals of Leicester NHS Trust, Leicester, UK

**Keywords:** multimorbidity, carer, frailty, physical activity, sedentary behaviour, intervention, older people

## Abstract

**Introduction:**

This scoping review mapped evidence on physical activity (including structured exercise) and sedentary behaviour interventions (interventions to reduce sedentary behaviour) in people living with both frailty *and* multiple long-term conditions (MLTCs) and their informal carers.

**Methods:**

Ten databases and grey literature were searched from 2000 to October 2023. Two reviewers screened studies and one extracted data. Results were shared with three stakeholder groups (*n* = 21) in a consultation phase.

**Results:**

After screening, 155 papers from 144 studies (1 ongoing) were retained. The majority were randomised controlled trials (86, 55%). Participants’ mean age was 73 ± 12 years, and 73% were of White ethnicity. MLTC and frailty measurement varied widely. Most participants were pre-to-moderately frail. Physical health conditions predominated over mental health conditions.

Interventions focused on structured exercise (83 studies, 60%) or combined interventions (55 studies, 39%). Two (1%) and one (0.7%) focused solely on habitual physical activity or sedentary behaviour. Adherence was 81% (interquartile range 62%–89%) with goal setting, monitoring and support important to adherence. Carers were only involved in 15 (11%) studies. Most interventions reported positive outcomes, primarily focusing on body functions and structures.

**Conclusions:**

A modest volume of evidence exists on multicomponent structured exercise interventions, with less focus on habitual physical activity and sedentary behaviour. Interventions report largely positive effects, but an updated systematic review is required. The field could be advanced by more rigorous characterisation of MLTCs, socioeconomic status and ethnicity, increased informal carer involvement and further evaluation of habitual physical activity and sedentary behaviour interventions.

## Key Points

- This scoping review maps the evidence on physical activity and sedentary behaviour interventions for individuals with frailty and multiple long-term conditions (MLTCs) and their caregivers.
- Eight (5%) of the studies were in low- to middle-income countries, including predominantly white, affluent participants and focusing on physical health conditions.
- Studies inclusive of more severely frail participants were lacking.
- Most studies focused on structured exercise, with limited attention to habitual physical activity or sedentary behaviour interventions.
- Fifteen (11%) studies included carers, primarily to enhance adherence and provide support.
- To advance the field, improved reporting, increased carer involvement and further evaluation of physical activity and sedentary behaviour interventions are required.

## Introduction

Globally, the number of people living with multiple long-term conditions (MLTCs, the co-existence of two or more chronic conditions) is growing [1], disproportionately impacting the socioeconomically disadvantaged [2, 3]. MLTCs are associated with adverse outcomes including reduced physical function, loss of independence and life participation [4–11]. Within this population, those also living with frailty, a ‘multidimensional syndrome of decreased physiological reserve leading to increased susceptibility to minor health stressors’, are particularly vulnerable to poor outcomes [12–14]. Data from the UK Biobank indicate that 7% of people living with at least two long-term conditions are also frail, rising to 18% in those living with at least four long-term conditions, whilst 72% of people living with frailty are multimorbid [14–16]. Physical inactivity (failure to achieve physical activity recommendations) and sedentary behaviour (seated/reclined behaviours requiring low energy expenditure) [17] are prevalent among people living with frailty and MLTCs, making the identification of effective interventions, and methods to increase the uptake of, and adherence to, these types of interventions clinically important [18–21] and a priority for research [22, 23].

Systematic reviews have examined structured exercise for people living with frailty [24, 25] and MLTCs [26] independently, with only one review in 2012, including both [27]. Whilst these reviews all indicate that multicomponent exercise programmes including strength and balance training are important for improved outcomes, exercise training may not be consistently feasible for people living with frailty *and* MLTCs due to treatment burden, ill-health, increased symptomology and functional variability [28–31]. Addressing sedentary behaviour and increasing habitual physical activity may provide a more acceptable and sustainable segue into structured exercise [32, 33]. Informal carer involvement may also be pivotal, influencing engagement and outcomes [34]. Given that frailty may affect people living with MLTCs at a younger age, a review inclusive of all age groups is warranted [15] as well as examining carers’ roles in these interventions.

## Aims and objectives

This scoping review mapped the evidence on physical activity (including structured exercise) and sedentary behaviour interventions (interventions to reduce sedentary behaviour) in people living with *both* frailty and MLTCs and their carers.

The following questions were addressed:

- What are the characteristics of the physical activity and sedentary behaviour interventions used?
- How have carers been included within intervention design, development and delivery?
- What approaches appear to contribute to improved outcomes and engagement?

## Methods

The scoping review methodology adhered to a six-stage process [35–38] and included stakeholder consultation. A detailed protocol has been published [39]. Reporting follows the PRISMA-ScR guidelines [40].

### Search strategy and selection criteria

The inclusion and exclusion criteria are outlined in Table 1. Studies were eligible if they included:

- Interventions with a physical activity or sedentary behaviour component, including multicomponent interventions.
- Participants predominantly living with frailty and MLTCs. Frailty was established using either an established frailty measure or validated functional proxy (see Appendix 1). We employed a hierarchical approach, whereby we recorded only the frailty measure in studies measuring both.

**Table 1:** Review inclusion and exclusion criteria

	Inclusion criteria		Exclusion criteria
**Study design**	Any study design examining interventions or intervention content (e.g. single-group pre–post-intervention, parallel-group design crossover or cluster, observational studies, intervention development papers, qualitative studies and process evaluations relating to interventions)		Non-English-language studies Studies in children or animals
**Population**	Adults aged ≥18 years		Presence of MLTCs not defined or <50% of the sample report MLTCs
	Living with both frailty and MLTCs	Multiple long-term conditions, co-existing conditions and single conditions where the majority of the samples were multimorbid. Included single long-term conditions: Anxiety Arthritis (osteo and rheumatoid) Asthma Cancer (solid organ, haematological and metastatic) Chronic kidney disease Chronic liver disease Chronic obstructive pulmonary disease Coronary artery disease Depression Heart failure HIV and AIDS Hyperlipidaemia Ischaemic heart disease Multiple sclerosis Obesity Osteoporosis Parkinson’s disease Peripheral artery disease Type 2 diabetes	Recognised measure of frailty or validated proxy not used
		Recognised, valid measure of frailty (e.g. clinical frailty scale, Fried frailty phenotype) **or** functional proxy with predefined cut-points indicating the presence of frailty: Modified Physical Performance Test (PPT) Balance Performance Oriented Mobility Assessment (BPOMA) Short Physical Performance Battery (SPPB) Timed Get-Up-and-Go test (TUAG) Gait speed test Sit to stand tests^*^: Sit to stand 30 s Sit to stand 60 s Sit to stand 5 repetitions Sit to stand 10 repetitions Handgrip strength^*^ Strength, assistance with walking, rising from a chair, climbing stairs and falls questionnaire (SARC- F questionnaire)	
**Concept (interventions)**	Interventions with a physical activity, exercise or sedentary behaviour focus, including multicomponent interventions		Studies of acute responses to sedentary behaviour, physical activity or exercise, including interventions of <1 week in duration
**Context**	Any setting		

^*^Published age-related norms were used to establish possible frailty.

*Abbreviations*: AIDS, acquired immune deficiency syndrome; BPOMA, Balance Performance Oriented Mobility Assessment; HIV, human immunodeficiency virus; MLTCs, multiple long-term conditions; mPPT, Modified Physical Performance Test; SPPB, Short Physical Performance Battery; SARC-F questionnaire, Strength, assistance with walking, rising from a chair, climbing stairs and falls questionnaire; TUAG, Timed Get-Up-and-Go test.

MLTCs were defined as the co-existence of two or more chronic conditions (physical or mental) [1]. Given the relatively recent use of MLTCs as a term, we included studies where the characteristics of the sample indicated the presence of MLTCs using comorbidity scores (e.g. Charlson Comorbidity Index) or where the majority of the sample (>50%) were living with MLTCs [26].

Ten databases, trials registries and grey literature (Appendices 2 and 3) were searched from January 2000 to October 2023. Ongoing trials were included where registered information was sufficient to confirm inclusion. Reviews were not included, but additional studies were identified via their references.

### Study selection

Studies were included through a systematic two-step process managed via Covidence (Veritas Health Innovation Ltd, Melbourne, Australia). Two independent reviewers screened abstracts and full texts. A third resolved discrepancies.

### Data charting

A standardised data charting tool using published checklists [41, 42] was used to extract data. Data charting included extraction of information regarding outcomes. Multiple reports from a single study were considered as one. Available protocol papers were reviewed for additional information.

### Stakeholder consultation

Consultation in scoping reviews is used to obtain a more comprehensive understanding of future research areas [43]. Results from the review were discussed with three stakeholder groups: people living with both frailty and MLTCs; informal carers; healthcare professionals (HCPs) and researchers. Groups convened separately to facilitate open discussion. Example questions used to guide the group discussion are included in Appendix 4. Facilitated by H.M.L.Y., meetings, either in person at accessible community centres or hospitals or virtually, were not audio-recorded. Instead, a co-facilitator (K.P. or M.T.) took notes and observed interactions. Ethical approval was provided by the University of Leicester (34246-hy162-ls:populationhealthscience, 3 November 2023). All participants provided written informed consent.

### Protocol deviations

Adjustments were made to data extracted (Appendix 5). The critical appraisal stage was removed. Data on outcomes were extracted from the abstract only.

### Data analysis

Descriptive statistics were used to summarise the data. Where means and standard deviation were not reported, they were estimated from medians and interquartile ranges (IQRs) [44]. Published guidance was used to summarise socioeconomic data [45]. Outcomes were categorised according to the WHO International Classification of Functioning, Disability and Health (ICF, Appendix 6) [46]. Qualitative data and data from the consultation process were independently summarised using basic content analysis [47]. Mixed-methods joint displays were used to integrate all data by objective [48].

## Results

### Selection of sources of evidence

**Figure 1:**
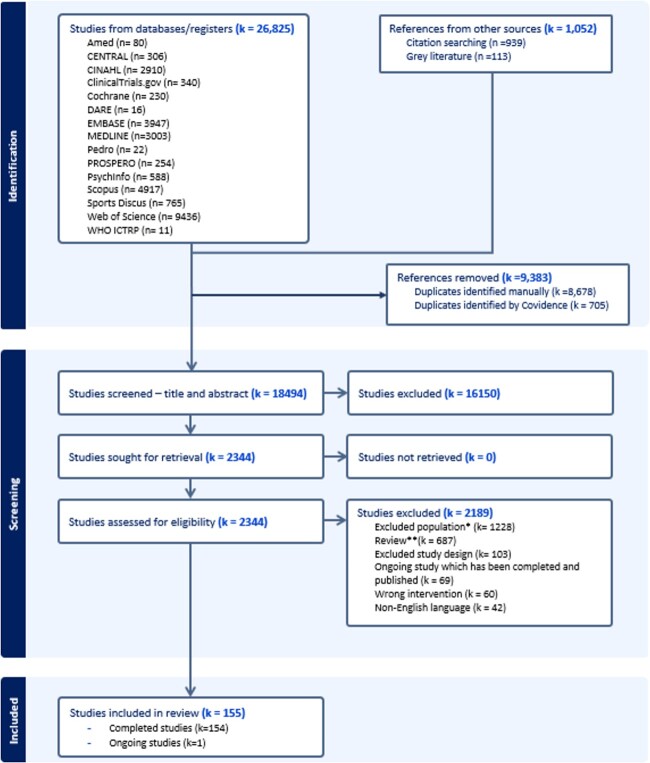
PRISMA flow diagram. ^*^Did not meet the inclusion criteria for MLTC, frailty or both, either because this information was not reported or because the population did not meet the identified cut-points or criteria. Other studies excluded due to this criterion included paediatric populations. ^**^Reviews were screened and relevant studies from their reference lists that were not already included were added (*k* = 939). These are highlighted in the references from other sources box.

From the initial 27 877 references, 155 papers from 143 completed and 1 ongoing study were included (Figure 1). Table 2 summarises the study characteristics, and full study details are included in Appendix 7

**Table 2:** Table of included papers

Summary characteristics		Number of studies (% or IQR where relevant)
**Country**	***High income***	133 (92%)
	***Low to middle income***	8 (5%)
	***Multi-country studies***	3 (2%)
Study design^*^	***Pre/post design***	11 (7%)
	***Parallel RCT***	85 (55%)
	***Pilot RCT***	13 (8%)
	***Cluster RCT***	4 (2%)
	***Controlled before and after study***	1 (0.6%)
	***Non-randomised experimental study***	11 (7%)
	***Designed-delay study***	1 (0.6%)
	***Observational study***	16 (10%)
	***Intervention development paper***	1 (0.6%)
	***Economic evaluation***	1 (0.6%)
	***Implementation study***	1 (0.6%)
	***Quality improvement/service evaluation***	2 (1%)
	***Qualitative***	7 (4%)
	***Ongoing study***	1 (0.6%)
Frailty assessment^**^	***Frailty measure***	51 (35%)
	***Functional proxy for frailty***	92 (64%)
Population^**^	***Older population with MLTCs***	70 (49%)
	***Index condition with MLTCs***	58 (40%)
	***MLTC inclusion criteria***	13 (9%)
	***ICU population with MLTCs***	2 (1%)
Intervention summary (all arms if applicable)^***^ ^**+**^	***Exercise***	82 (58%)
	***Habitual physical activity***	2 (1%)
	***Sedentary behaviour intervention***	1 (0.7%)
	***Combined intervention (at least one movement behaviour with additional interventions)***	55 (39%)
Intervention provider ^***^ ^**♦**^	***Physiotherapist***	71 (51%)
	***Exercise professional***	38 (27%)
	***Nurse***	12 (8.6%)
	***Researcher***	9 (6.4%)
	***Health worker***	8 (6%)
	***Doctor***	7 (5%)
	***Volunteer***	5 (3.6%)
	***Dietitian***	2 (1.4%)
	***Occupational therapist***	2 (1.4%)
	***Social worker***	1 (0.7%)
	***Psychologist***	1 (0.7%)
	***Not reported***	16 (11%)
**Carer involvement**	***Yes***	15 (10%)

^*^Presented as number of papers as some studies report multiple papers with different foci (e.g. trial and economic evaluation). ^**^Completed studies only. ^***^Completed studies excluding qualitative studies. ^+^Data on frequency, intensity, time, duration, decision rules for starting levels, progression and tailoring are summarised in detail in Appendix 13. ^♦^Some studies report multiple providers.

### Study characteristics

Most papers were randomised controlled trials (RCTs) (85, 55%) [49–133], observational research (16, 10%) [28, 134–148] or pilot/feasibility studies (13, 8%) [29, 149–160]. Seven (4%) utilised a qualitative approach [161–167], and five (3%) used mixed methods [29, 149, 152, 160, 168]. Of the papers that included a qualitative approach or component (12, 8%), 10 (83%) focused on the perspectives of the person living with frailty and MLTCs [29, 149, 152, 154, 160–165], and 2 (17%) the HCP [166, 167]. No studies directly explored the views of carers.

Only eight studies (5%) were conducted in low- and middle-income countries [71, 73, 81, 100, 107, 108, 123, 169]. A total of *n* = 25 745 participants were included. Study sample sizes ranged from 10 to 4859, with a median of 75 participants (IQR 43–138) per study. Participants’ mean age was 73 ± 12 years, and 13 679 (60%) were female. Ninety-nine studies (69%) did not report ethnicity [28, 50, 52, 53, 56, 58–63, 69–74, 76–81, 83, 85, 86, 89–94, 96–101, 103, 104, 106–116, 120–122, 124–126, 131–138, 140, 142, 143, 145, 147, 149, 152, 158, 161, 163, 168–185]. Within the remaining 44 (30%) studies, the reporting of ethnicity was variable [29, 49, 54, 55, 57, 67, 68, 75, 82, 84, 87, 88, 95, 102, 117–119, 123, 127–130, 139, 141, 144, 150, 151, 153–157, 159, 160, 162, 186–195]. Where it was possible to summarise these data (40 studies), 73% were White, 33% Chinese, 18% Black, 13% Hispanic and 10% Asian. Data on socioeconomic status were reported in 52 (36%) studies (Appendix 8) [49, 50, 53, 54, 63, 67, 68, 70, 73–75, 77, 84, 87, 92, 100, 102–104, 106, 108, 111, 117, 118, 123, 125, 129–133, 144, 150, 160, 162, 163, 169–171, 174, 175, 177–179, 182, 186, 187, 191, 193–195]. Participants were typically well educated and affluent.

#### Multiple long-term condition measurement

Thirteen studies (9%) used the presence of MLTCs as an inclusion criterion [49, 53, 71, 74, 93, 104, 109, 119, 141, 145, 182, 186, 194]. Of these, eight framed MLTCs around a physical health index condition together with one other [53, 71, 74, 93, 119, 141, 145, 186]. The remaining studies were included because the majority of the sample were living with MLTCs based on their baseline characteristics. Of these, 70 (49%) focused older populations [51, 52, 54, 57, 59, 60, 62, 64–66, 68–70, 72, 75, 77, 80–82, 84, 87, 89–92, 94, 95, 97, 100–102, 104–106, 110–112, 116, 118, 120, 122–127, 132, 134, 136, 137, 146, 147, 149, 151, 152, 158, 160, 161, 163, 168, 169, 176, 177, 179, 180, 185, 187, 191, 192, 195, 196], 58 (40%) on an index condition [28, 29, 50, 55, 56, 58, 61, 63, 67, 73, 76, 78, 79, 83, 85, 86, 88, 89, 96, 98, 99, 103, 107, 113–115, 117, 121, 128–131, 135, 138–140, 142–144, 150, 154–157, 162, 171–173, 175, 178, 181, 183, 184, 188–190, 193] and 2 (1%) on people in intensive care [153, 159]. The frequency of included index conditions is displayed in Appendix 9. These studies used a range of methods to quantify MLTCs (Appendix 10).

#### Frailty measurement

Fifty-one studies (36%) measured frailty using a validated measure [28, 29, 51, 62, 63, 65, 68, 69, 74, 77, 78, 80, 81, 89, 99, 101, 106, 109, 111, 113, 117, 119, 122, 123, 132–134, 136–138, 142, 144–147, 151–154, 156, 160, 161, 174, 177, 179, 180, 182, 184, 185, 188, 190], with slightly more characterising physical rather than general frailty. Most participants in these studies were pre-to-moderately frail (Appendix 11). Ninety-two studies (64%) used a functional proxy indicative of frailty (Appendix 12) [49, 50, 52–61, 64, 66, 67, 70–73, 75, 76, 79, 82–88, 90–98, 100, 102–105, 107, 108, 110, 112, 114–116, 118, 120, 121, 124–131, 135, 139–141, 143, 149, 150, 155, 157–159, 162, 163, 168, 169, 171–173, 175, 176, 178, 181, 183, 186, 187, 189, 191–195].

### Characteristics of interventions

#### Components

Across all study designs, except qualitative studies which did not provide specific information of intervention characteristics [161–163], 82 studies (58%) evaluated a structured exercise intervention [28, 29, 49–56, 59–61, 63, 64, 68, 70, 71, 74, 76, 79, 81, 83, 85, 88, 90, 91, 95–103, 105, 106, 108, 109, 112, 114–116, 118, 120–122, 124, 127, 135, 137–139, 141, 143, 145, 149–154, 156–159, 168–172, 175, 178, 180, 183, 184, 186, 188–190, 192], 2 (1%) habitual physical activity [110, 147] and 1 (0.7%) sedentary behaviour as stand-alone interventions [123]. Fifty-five (39%) studies included a combination of interventions [57, 58, 62, 65–67, 69, 72, 73, 75, 77, 78, 80, 82, 84, 86, 87, 89, 92–94, 104, 107, 111, 113, 117, 119, 125, 126, 128–134, 136, 140, 142, 144, 155, 160, 173, 174, 176, 177, 179, 181, 182, 187, 191, 193–195], of which at least one component targeted physical activity or sedentary behaviour. Within these combined interventions, 15 (28%) included a component that focused on increasing habitual physical activity [58, 65, 72, 77, 82, 92, 104, 111, 119, 125, 126, 155, 191, 193, 194] and 1 (2%) a component that addressed sedentary behaviour [126]. The remaining studies all incorporated a structured exercise component.

**Figure 2:**
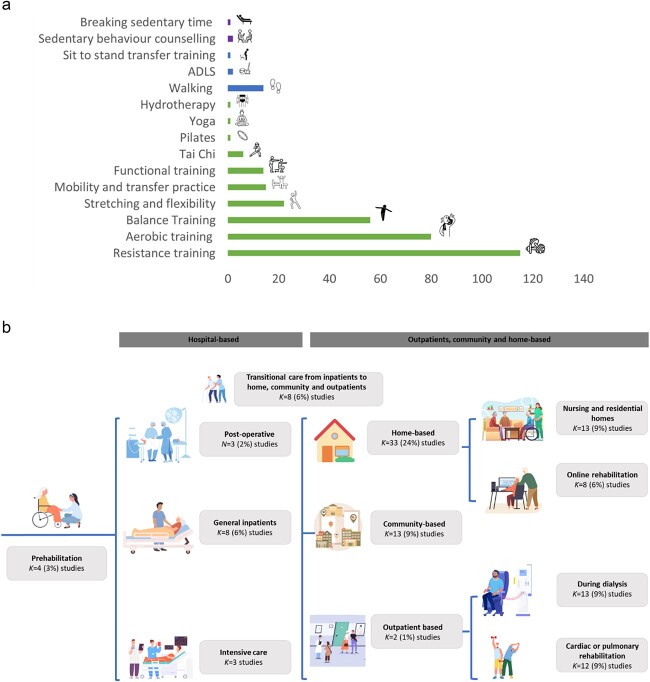
(a) Frequency of the intervention components within the included studies. Exercise intervention components are highlighted in green, habitual physical activity components in blue and sedentary behaviour interventions in purple. *Abbreviations*: ADLS, activities of daily living. (b) Settings and timings in which the interventions took place. Interventions across all arms are included.

Figure 2a outlines the components included within the interventions. Within all the studies that included structured exercise (136, 98%) [28, 29, 49–109, 111–118, 120–122, 124–145, 149–160, 168–184, 186–192, 194, 195], the majority (97, 71%) included multicomponent exercises [158, 50, 51, 54–57, 60–63, 65–69, 72–74, 126–131, 134–137, 149, 151, 155, 159, 160, 170–174, 184, 202]. Where reported, interventions that included habitual physical activity focused on walking (12, 9%) [60, 71, 93, 108, 125, 134, 135, 139, 154, 161, 190, 191], activities of daily living (1, 1%) [162] or a combination of these (2, 1%) [81, 185]. The two studies that addressed sedentary behaviour interrupted sedentary time with standing balance exercises, and provided personalised self-management guidance and telephone support [159, 162].

Qualitative data from four studies indicated that people living with frailty and MLTCs viewed physical activity as everyday ‘work’ (e.g. gardening, activities of daily living or community roles) [29, 192, 194, 195]. These were viewed as purposeful, meaningful and often led people to believe they were doing ‘enough’ to preserve their health and independence. The importance of strength training were only fleetingly mentioned in three studies [29, 63, 169]. No studies explored experiences of, or attitudes towards, sedentary behaviour interventions.

#### Intervention delivery

All studies, except one that focused on HCP training [86], targeted the intervention towards people living with frailty and MLTCs. The majority of interventions were delivered by physiotherapists (71, 51%) [28, 29, 49, 51, 55, 58, 62, 63, 65, 68, 72, 78, 80–89, 91, 96–98, 100, 103, 104, 107, 110, 113–116, 118, 122, 124–126, 133, 136, 137, 139, 140, 142, 144, 146, 149, 150, 155, 157–159, 161–167, 172–175, 178, 179, 182, 183, 189, 191] or exercise professionals (38, 27%) [29, 52, 53, 56, 57, 59, 60, 63, 71, 75, 79, 88, 90, 92–96, 108, 111, 119, 120, 124, 129, 130, 135, 138, 141, 143, 144, 148, 151, 156, 160, 168–170, 185]. Settings and timings of the interventions are displayed in Figure 2b. Qualitative data from six studies indicated that home-based interventions reduce burden, travel time and costs, and enhance flexibility [29, 137, 193, 195–197]. They were also preferable for those with limited physical ability, or high symptom burden, ensuring consistency and adaptability around the individual’s health and priorities [195, 196]. Group and in-centre options were viewed more favourably when the frequency and duration of the programme could be negotiated [193]. Incorporating opportunities to see the benefits of the intervention reduced participants’ perception of the delivery mode’s burden [103, 137, 193, 195, 196].

#### Prescription and tailoring

Prescription, progression and tailoring were highly heterogeneous across interventions (Appendix 13). Tailoring was evident in 56 (41%) studies most commonly in response to participants’ abilities (38, 27%) [29, 53, 68, 81, 84, 86, 98, 100, 107, 108, 110, 112–114, 117–119, 129, 131, 134, 137, 142, 145, 147, 148, 151, 160, 163, 165, 170, 174, 176, 177, 182, 183, 189, 200, 201] and symptoms (13, 9%) [51, 82–84, 89, 93, 107, 150, 153, 157, 170, 182, 189] and, to a lesser extent, co-existing conditions (5, 4%) [83, 84, 98, 116, 151] and frailty status (3, 3%) [68, 80, 176].

Seven qualitative or mixed-methods studies highlighted the importance of tailoring according to the same factors elucidated in the quantitative studies [29, 192, 194–198]. People undertook their own adaptations to activity in response to their changing abilities [192, 193]. Without appropriate guidance, these included avoidance and ‘slowing down’ [194]. Those living with mental health conditions were specifically identified as requiring a tailored approach, as these were perceived as hindering in-person group participation and internal motivation [196, 197]. Periods of increased symptomatology also required tailoring, including education on interpreting symptoms [29, 195], tracking the impact of symptoms on activity [195], avoiding unhelpful attitudes or avoidant behaviours [196], and maintaining appropriate activity during periods of increased symptom burden [195, 196].

### Inclusion of informal carers

Only 15 (10%) studies included carers [69, 78, 86, 91, 129, 133, 145, 155, 159, 176, 188, 191, 193, 195, 200]. Eight (5%) involved them in the intervention, often according to the preference of the person living with frailty and MLTCs [91, 133, 155, 188, 191, 193, 195, 200]. Four studies (3%) actively enrolled carers as participants or provided them with a specific intervention [91, 188, 191, 195]. Demographic data for carers were largely absent. One study reported that carers provided a median of 9.25 hours of care per week, and had a Zarit carer burden score of 5.6, indicating a low level of labour experienced by the included carers [91]. Seven studies (5%) included carers to enhance adherence [69, 78, 86, 129, 145, 159, 176]. Qualitative data revealed that whilst informal carer involvement could increase adherence (Appendix 14), this needed to be negotiated to maintain participants’ sense of independence and preserve positive relationships [193].

### Engagement and adherence

Forty studies (29%) did not report adherence [50, 64, 69, 70, 74, 75, 81, 82, 86, 91, 93, 94, 110, 113, 121, 123, 125, 127, 132–134, 136, 142, 144, 146, 149, 151, 154, 157, 160, 165, 173, 174, 176–178, 180–182, 185]. Of the remaining studies, where data could be summarised, the median percentage adherence was 81% (IQR 62–89). Qualitative data from six studies described how deciding to participate involved weighing up the potential to maintain or improve independence and wellbeing with the risk of adverse events, including falls, exacerbations of their MLTCs and symptoms, and injury [29, 63, 192, 193, 195, 198]. One study also highlighted that informal carers had influential concerns regarding safety [195].

**Figure 3:**
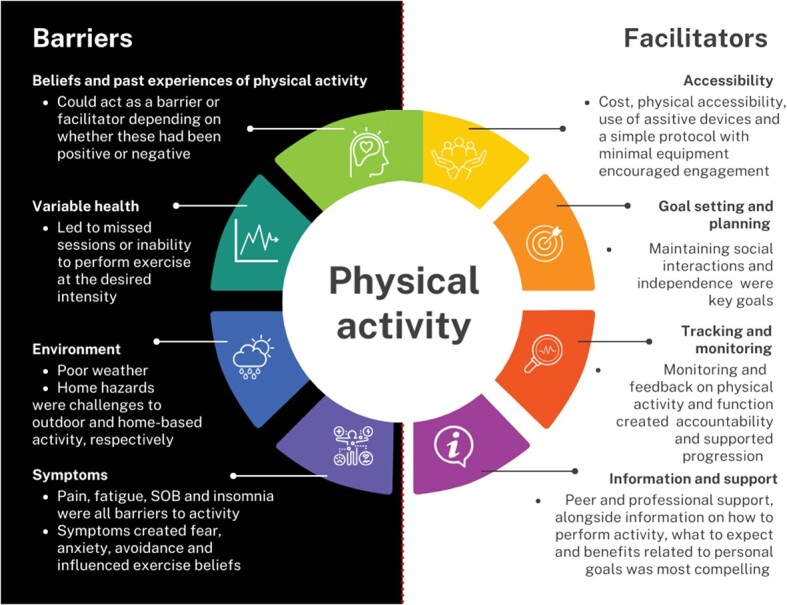
Barriers and facilitators to physical activity in people living with frailty and MLTCs, drawn from qualitative data [29, 103, 137, 169, 192–198].

Barriers and facilitators to initial engagement with, and ongoing adherence to, habitual physical activity and exercise taken from qualitative data are outlined in Figure 3 [29, 103, 137, 169, 192–198]. No qualitative studies examined sedentary behaviour interventions. Seven studies emphasised setting goals related to maintaining social interactions and independence [29, 103, 192, 194, 196–198]. Linking motivation to achieve these goals with physical activity was key to ongoing adherence [169, 192–197]. Monitoring and feedback on physical activity and functional ability was important to increase knowledge, accountability and progress [169, 195, 196]. However, feedback required care, as some people living with frailty and MLTCs were not going to improve, but maintenance, slowing decline and achieving personal goals remained highly valued [194].

Peer support provided the opportunity for shared experience, camaraderie and friendly competition [29, 193, 196], whilst professional support was seen as key for safety and motivation. Effective peer support centred around grouping people of similar abilities together [29, 193, 196]. Effective professional support centred around developing a consistent, positive therapeutic relationship [103, 137, 195, 196].

Past experience and beliefs about activity served as both a barrier and facilitator, depending upon the individual. Those who had been active lifelong were more likely to seek alternative activity as their abilities changed, to continue to enjoy a challenge and be more likely to sustain their activity, even if their health fluctuated [192, 194, 196]. Recent adopters engaged in physical activity to achieve a personal goal, rather than for its own sake [194, 196].

### Reported effects

Across the papers reporting outcomes (140, 91%), a total of 333 separate outcomes were identified, with a median of 7 (IQR 4–10) measures per study. Appendix 15 provides an overview of intervention effectiveness. Overall, the majority measured outcomes relating to the body functions and structures domain of the ICF, predominantly relating to muscle and exercise capacity. Balance, mobility, flexibility, dexterity and physical function were the most frequently measured for the activity and participation domain, whilst healthcare utilisation was the most measured environmental domain. Positive effects were reported across most domains, except for impacts on medication and carer health. Qualitative data highlighted opposing views about the benefits, ranging from the belief in an inevitable decline with age to the conviction that physical activity could support adaptation to a ‘changed self’, improve resilience and prevent disease and frailty progression [103, 193–195].

### Ongoing studies

One ongoing study was eligible [199]. This mixed-methods study will use interviews and survey data to describe health and lifestyle factors in people living with frailty and MLTCs, and their attitudes to exercise.

### Consultation

Twenty-four stakeholders were invited to, and 21 (87%) attended, a consultation meeting. Seven (33%) were people living with frailty and MLTCs, *n* = 6 (29%) were informal carers and *n* = 8 (38%) were healthcare professionals or researchers. Full characteristics are included within Appendix 16. Key gaps discussed are summarised in detail, alongside the primary review findings within a joint display (Appendix 17).

## Discussion

This review indicates that the majority of evidence comes from RCTs in high-income countries with predominantly white, socioeconomically advantaged populations. The presence of MLTCs was poorly characterised and measurement varied widely. An emphasis on structured multicomponent exercise was evident. Carer involvement and outcomes related to carer health were under-represented. Outcomes focused primarily on body functions and structures and, to a lesser extent, activity and participation appeared to indicate that interventions are effective.

The impact of MLTCs and frailty, especially on socioeconomically disadvantaged [10, 15, 203, 204] and ethnic minority populations [205, 206] in low- and middle-income countries [207, 208], underscores the need for consistent reporting of these characteristics and better representation in future research. It also has implications for the types of interventions evaluated. Interventions requiring expensive equipment, significant space or increasingly associated with privilege (e.g. yoga and Pilates) [209] may not be accessible to under-represented populations. Similarly, interventions that rely on walking should be mindful of the potential to perpetuate inequity in neighbourhoods with lower levels of walkability [210].

The presence of MLTCs was not as well characterised as frailty, perhaps reflecting an emerging interest in this area. Whilst convenient for research purposes, a continuing focus on chronological age and specific conditions may fail to recognise the heterogeneity within multimorbid populations and miss opportunities to develop targeted and effective interventions for those most at risk. Future studies may make better use of the identification of clusters of long-term conditions associated with frailty and poorer outcomes, and those that might best respond to a physical activity or sedentary behaviour intervention, enhancing effectiveness [211]. The lack of inclusion of mental health conditions is notable given that people living with severe mental illness are more likely to live with MLTCs [212, 213] and frailty [214], and face poorer outcomes [213–217]. Recognising the pronounced physical inactivity and sedentary behaviour in this population [213], interventions addressing these behaviours could positively impact both physical and mental health outcomes [213]. Further research is needed to establish how interventions should be tailored for this population [213, 218].

Carer involvement was under-reported, and there appeared to be a dearth of research including carers. Whilst there are many positive effects of caregiving [219, 220], it is also associated with a range of negative impacts [219–222]. Physical activity and sedentary behaviour interventions that only involve caregivers to enhance adherence may inadvertently add to this burden. This review indicates negative outcomes relating to carer health, and further research is needed to better understand this potential issue. Carers also face a 16% higher risk of developing MLTCs than non-carers [223], and caring for a spouse living with frailty is an independent risk factor for frailty among older adults [224, 225]. Given that carers are more likely to be physically inactive [226], interventions targeting carers are also important for their own health and wellbeing. Such interventions improve carer psychological wellbeing [227–230], physical health [229, 230], sleep [228], habitual physical activity [228] and quality of life [228].

Caregivers and care recipients influence each other’s behaviours [231–234]. Future research that considers this reciprocal influence, and the diverse nature of caring relationships, is needed. Physical activity and sedentary behaviour interventions should be co-designed in partnership with both people living with frailty and MLTCs *and* carers. Areas of particular importance to consider include ensuring that undue carer burden is avoided, that carers also benefit from the programme and that carer participation is managed flexibly to support different relationships and needs. This review also emphasises the need to improve reporting around carer involvement, including information regarding their demographics, their relationship to the person with MLTCs and frailty, the nature of caring provided (e.g. mean number of hours of care provided and markers of carer burden) and the nature and purpose of their involvement.

Structured exercise, often incorporating multicomponent elements (resistance, aerobic and balance training), predominated across included studies. Resistance training is crucial for addressing the sarcopenia associated with frailty and MLTCs [235]. Used alongside aerobic exercise, it enhances cardiorespiratory fitness [235] and, when combined with challenging balance training, reduces the risk of falls [235]. Despite this, there seemed to be relatively little focus on resistance training within the qualitative studies identified in this review, signalling a potential lack of emphasis on this component among people living with frailty and MLTCs and HCPs, a finding consistent with studies in older populations [236, 237]. Since the completion of this review, the first paper from the included ongoing study has been published [199, 238], uncovering a lack of understanding regarding resistance training and reporting similar barriers and facilitators [238].

The studies that examined the effects of both habitual physical activity and sedentary behaviour prioritised walking and substituting sitting with standing balance exercises and counselling. Habitual activity and sedentary behaviour interventions may represent important management strategies for people living with frailty and MLTCs, and for preventing the development of further additional long-term conditions [239]. They may prepare people to move towards more structured exercise [32] and to increase consistency, given the preference for home-based interventions in this population. Our review also suggests that people living with frailty and MLTCs view habitual physical activity as ‘everyday work’. Given this, incorporating simple, brief, functional resistance exercises into daily routines, and using these to break sedentary behaviour, may be a meaningful treatment strategy, particularly in a population for whom increased walking might pose increased risks of falls [240, 241]. Future research could explore the effectiveness of this approach. Qualitative studies investigating factors influencing sedentary behaviour would also aid intervention design.

Both the findings of this review, specifically the qualitative results relating to engagement adherence and outcomes, and the consultation process, highlight the potential role for shared decision-making (SDM) in physical activity and sedentary behaviour interventions [242]. SDM may improve both healthcare experience and adherence [242] and is well suited to an intervention where many options exist and the risks and benefits of these behaviours may be viewed and valued differently by different individuals. SDM may support the identification of meaningful goals and a better understanding of the priorities and preferences of people living with frailty and MLTCs, and those who care for them. It may also support the open and unbiased discussion of the risks and benefits of different movement behaviours, and of maintaining the status quo, supporting people to actively co-design an activity programme that is right for them. To date, there has been relatively little exploration of SDM in relation to physical activity and sedentary behaviour [243–245], and this may be a beneficial area for future research.

Whilst this scoping review cannot draw conclusions about the effectiveness of interventions, the broadly positive effects align with similar reviews of exercise in this population [27], and MLTC [26, 229] and frail populations [24] in isolation. Given the time elapsed since the last systematic review of exercise interventions in people living with both MLTC and frailty [27], an update is recommended. Such a review could also examine maintenance of effects following withdrawal of intervention. Future studies should prioritise measuring domains important to people living with frailty and MLTCs from core outcome sets [246, 247]. Furthermore, adequate reporting of the presence and severity of frailty and MLTCs in trials of single conditions would also inform the care of people living with frailty and MLTCs within future synthesis. Finally, considering long-term maintenance of behaviours following cessation of the intervention in future studies would help establish the clinical utility of these interventions.

The field of MLTC research is rapidly evolving and this scoping review provides a comprehensive and contemporary overview of the available evidence for physical activity and sedentary behaviour interventions in those who are also frail. Despite its strengths, there are several limitations. Following the publication of our protocol, an international consensus study provided further guidance on conditions to include within MLTC research [248]. Most study samples did not exclusively comprise individuals living with frailty or MLTCs. In the absence of guidance, we included studies where the characteristics of the sample indicated that the majority were living with frailty or MLTCs. A higher percentage cut point, as has been applied in other studies, may have led to more focused results [26]. The use of validated functional proxies for frailty ensured the inclusion of relevant papers that did not solely focus on chronologically older individuals in which frailty tools have typically been validated. Despite this, they may lack specificity for frailty [249]. Some frailty measures (e.g. the Frailty Index) include long-term health conditions. Consequently, there may be some overlap between the concepts of frailty and MLTC within studies using these measures. In response to the large volume of eligible studies identified, we removed the critical appraisal stage and outcome data were extracted from the abstracts. These modifications precluded an assessment of study quality and may have meant that only some outcomes were reported. Finally, we aimed to scope a wide range of study designs, and included within this quality improvement and service evaluations, which may have been conducted with less methodological rigour than other approaches.

## Conclusions

Modest evidence exists on exercise for frailty and MLTCs, with less focus on habitual activity and sedentary behaviour interventions. Research should address this gap, and target clusters of MLTCs, crucial outcomes and examine the optimal involvement of informal carers, in more diverse populations and geographical settings. An updated systematic review would also provide an assessment of the effectiveness of these interventions.

## Supplementary Material

aa-24-0271-File002_afae255
