# Supplementary material for: Physical activity and sedentary behaviour interventions for people living with both frailty and multiple long-term conditions and their informal carers: a scoping review and stakeholder consultation
Source: Age Ageing. 2024 Nov 19;53(11):afae255. doi: 10.1093/ageing/afae255 (PMC11574057; doi:10.1093/ageing/afae255)
Supplement: aa-24-0271-File002_afae255 [file aa-24-0271-file002_afae255.docx]

**Contents**

Appendix 1. Functional measures which are recognised proxy measures of frailty, and published cut-offs indicative of frailty.

Appendix 2. Sources searched in the scoping review

Appendix 3: Medline search strategy

Appendix 4. Semi-structured topic guide for scoping review consultation group

Appendix 5. Data extraction items originally outlined in the protocol, and those retained in the refined final data extraction tool.

Appendix 6. Outcome measures used within included studies categorised according to the domains of the World Health Organisation International Classification of Functioning and Disability (WHO-ICF).

Appendix 7. Characteristics of included studies

Appendix 8. Markers of socioeconomic status within the included studies.

Appendix 9. Frequency of the index conditions reported within the included studies.

Appendix 10. Measures of co-morbidity used within the included studies, and mean values, where reported.

Appendix 11. Measures of frailty used within the included studies, and mean values (where reported) within these studies.

Appendix 12. Functional proxies for frailty used within the included studies and mean values, where reported.

Appendix 13. Details of the prescription of included interventions.

Appendix 14. Ways in which informal carer support enhances physical activity engagement and adherence

Appendix 15. Reported outcomes of interventions, organised according to ICF categories.

Appendix 16. Characteristics of stakeholder group participants.

## Appendix 17. Results of the consultation process and ioint display combining key findings from the review with key themes from stakeholder engagement.

**Appendix 1. Functional measures which are recognised proxy measures of frailty, and published cut-offs indicative of frailty**

| **Function test** | **Published cut points for the identification of frailty** |
| --- | --- |
| Modified Physical Performance Test (mPPT) | Score range: 0-36^250^:   - Not frail: 32-36 - Mild frailty: 25-32 - Moderate frailty: 17-24 - Dependent: <17 |
| Balance Performance Oriented Mobility Assessment (BPOMA) | Score >19^250^ |
| Short Physical Performance Battery (SPPB) | A score of ≤ 7 is indicative of frailty^251^ |
| Timed Get-Up-and-Go test (TUAG) | A score of ≥ 9 seconds^252^ |
| Gait speed test | A gait speed of 0.8m/s is indicative of frailty  Taking more than 5 seconds to walk 4m^249^ |
| Sit to stand tests:   - Sit to stand 10 seconds - Sit to stand 30 seconds - Sit to stand 60 seconds - Sit to stand 5 repetitions - Sit to stand 10 repetitions | Dependent upon the type of sit-stand test used:   - ≥ 10 seconds for the five times sit stand^252^ - Lower than published reference values stratified by age and gender |
| Handgrip strength | Scores within the lowest quartile, stratified by sex^253^ |
| Strength, assistance with walking, rising from a chair, climbing stairs, and falls (SARC- F) questionnaire | Score of ≥ 4^254^ |

Abbreviations: BPOMA, Balance Performance Oriented Mobility Assessment; mPPT, Modified Physical Performance Test; SARC- F questionnaire, Strength, assistance with walking, rising from a chair, climbing stairs, and falls questionnaire; SPPB, Short Physical Performance Battery; TUAG, Timed Get-Up-and-Go test.

**Appendix 2. Sources searched in the scoping review**

For systematic reviews: Cochrane; PROSPERO; Database of Abstracts of Reviews of Effects.

For published research: MEDLINE; EMBASE; Cumulative Index to Nursing and Allied Health (CINAHL); Web of Science; Sports Discus; PsycINFO; Pedro; Allied and Complementary Medicine; Cochrane Central Register of Controlled Trials (CENTRAL); Scopus.

For grey literature: internet searching (eg, Google Scholar), BIOSIS previews, the Index to Scientific and Technical Proceedings.

For ongoing trials: CENTRAL; US National Institutes of Health Ongoing Trials Register (ClinicalTrials.gov) and the WHO International Clinical Trials Registry Platform.

**Appendix 3: Medline search strategy**

| 1 | exp comorbidity/ |
| --- | --- |
| 2 | Comorbid*.mp. |
| 3 | co-morbid*.mp. |
| 4 | exp multimorbidity/ |
| 5 | Multimorbid*.mp. |
| 6 | multi-morbid*.mp. |
| 7 | multidisease*.mp. |
| 8 | multi-disease*.mp. |
| 9 | exp multiple chronic conditions/ |
| 10 | “multiple chronic conditions”.mp. |
| 11 | poly-morbidit*.mp. |
| 12 | polymorbidit*.mp. |
| 13 | poly-patholog*.mp. |
| 14 | polypatholog*.mp. |
| 15 | pluripatholog*.mp. |
| 16 | pluri-patholog*.mp. |
| 17 | multipatholog*.mp. |
| 18 | multi-patholog*.mp. |
| 19 | multicondition*.mp. |
| 20 | multi-condition*.mp. |
| 21 | exp Syndemic/ |
| 22 | syndemic.mp. |
| 23 | "multiple health".mp. |
| 24 | "multiple chronic".mp. |
| 25 | "multiple long-term".mp. |
| 26 | condition*.mp. |
| 27 | ill*.mp. |
| 28 | disorder*.mp. |
| 29 | disease*.mp. |
| 30 | 23 or 24 or 25 |
| 31 | 26 or 27 or 28 or 29 |
| 32 | 30 and 31 |
| 33 | exp Diabetes Mellitus, Type 2/ |
| 34 | “diabetes type 2”.mp. |
| 35 | “diabetes type II”.mp. |
| 36 | “early onset diabetes”.mp. |
| 37 | (diabetes adj3 type 2).mp. |
| 38 | (diabetes adj2 type ii).mp. |
| 39 | (diabetes adj type two).mp. |
| 40 | T2DM.mp. |
| 41 | T2D.mp. |
| 42 | 33 or 34 or 35 or 36 or 37 or 38 or 39 or 40 or 41 |
| 43 | exp Hypertension/ |
| 44 | exp Blood Pressure/ |
| 45 | “Blood Pressure”.mp. |
| 46 | hypertens*.mp. |
| 47 | 43 or 44 or 45 or 46 |
| 48 | exp Heart Diseases/ |
| 49 | exp Heart Failure/ |
| 50 | exp Cardiovascular Diseases/ |
| 51 | exp Coronary Disease/ |
| 52 | cardiac.mp. |
| 53 | heart.mp. |
| 54 | cardiovascular.mp. |
| 55 | coronary.mp. |
| 56 | 52 or 53 or 54 or 55 |
| 57 | 31 and 56 |
| 58 | 48 or 49 or 50 or 51 or 57 |
| 59 | exp Vascular Diseases/ |
| 60 | exp Carotid Artery Diseases/ |
| 61 | cerebrovascular.mp. |
| 62 | vascular.mp. |
| 63 | Carotoid*.mp. |
| 64 | Arter*.mp. |
| 65 | 61 or 62 or 63 or 64 |
| 66 | 31 and 65 |
| 67 | exp Cerebrovascular Disorders/ |
| 68 | 59 or 60 or 66 or 67 |
| 69 | exp Asthma/ |
| 70 | asthma*.mp. |
| 71 | 69 or 70 |
| 72 | exp Pulmonary Disease, Chronic Obstructive/ |
| 73 | COPD.mp. |
| 74 | “chronic obstructive pulmonary disease”.mp. |
| 75 | 72 or 73 or 74 |
| 76 | exp Hyperlipidemias/ |
| 77 | exp Hypercholesterolemia/ |
| 78 | exp Hypertriglyceridemia/ |
| 79 | hyperlipid*em*.mp. |
| 80 | hypercholesterol*emia$.mp. |
| 81 | hypertriglycerid*emia*.mp. |
| 82 | 76 or 77 or 78 or 79 or 80 or 81 |
| 83 | exp Arthritis/ |
| 84 | exp Arthritis, Rheumatoid/ |
| 85 | exp Osteoarthritis/ |
| 86 | arthritis.mp. |
| 87 | “rheumatoid arthritis”.mp. |
| 88 | osteoarthritis.mp. |
| 89 | 83 or 84 or 85 or 86 or 87 or 88 |
| 90 | exp Depression/ |
| 91 | exp Anxiety/ |
| 92 | depression.mp. |
| 93 | anxiety.mp. |
| 94 | 90 or 91 or 92 or 93 |
| 95 | exp Neoplasms/ |
| 96 | malignan*.mp. |
| 97 | cancer.mp. |
| 98 | 95 or 96 or 97 |
| 99 | exp HIV/ |
| 100 | exp Acquired Immunodeficiency Syndrome/ |
| 101 | “acquired immunodeficiency syndrome”.mp. |
| 102 | AIDS.mp. |
| 103 | HIV.mp. |
| 104 | 99 or 100 or 101 or 102 or 103 |
| 105 | exp Kidney Failure, Chronic/ |
| 106 | exp Kidney Diseases/ |
| 107 | exp Renal Insufficiency/ |
| 108 | renal.mp. |
| 109 | kidney.mp. |
| 110 | 108 or 109 |
| 111 | 31 and 110 |
| 112 | 105 or 106 or 107 or 111 |
| 113 | exp Liver Diseases/ |
| 114 | liver.mp. |
| 115 | 31 and 114 |
| 116 | 113 or 115 |
| 117 | exp Osteoporosis/ |
| 118 | osteoporosis.mp. |
| 119 | 117 or 118 |
| 120 | exp Obesity/ |
| 121 | obes*.mp. |
| 122 | 120 or 121 |
| 123 | exp Multiple Sclerosis/ |
| 124 | multiple sclerosis.mp. |
| 125 | "MS".mp. |
| 126 | 123 or 124 or 125 |
| 127 | exp Parkinson Disease/ |
| 128 | “parkinsons disease”.mp. |
| 129 | "PD".mp. |
| 130 | 127 or 128 or 129 |
| 131 | peripheral artery disease.mp. |
| 132 | exp Peripheral Arterial Disease/ |
| 133 | 131 or 132 |
| 134 | co-occur*.mp. |
| 135 | cooccur*.mp. |
| 136 | concurrent.mp. |
| 137 | coexist*.mp. |
| 138 | co-exist*.mp. |
| 139 | 134 or 135 or 136 or 137 or 138 |
| 140 | 31 and 139 |
| 141 | 1 or 2 or 3 or 4 or 5 or 6 or 7 or 8 or 9 or 10 or 11 or 12 or 13 or 14 or 15 or 16 or 17 or 18 or 19 or 20 or 21 or 22 or 32 |
| 142 | 42 or 47 or 58 or 68 or 71 or 75 or 82 or 89 or 94 or 98 or 104 or 112 or 116 or 119 or 122 or 126 or 130 or 133 |
| 143 | 140 and 142 |
| 144 | 141 or 143 |
| 145 | exp Frail Elderly/ |
| 146 | exp Frailty/ |
| 147 | frail*.mp. |
| 148 | prefrail.mp. |
| 149 | pre-frail.mp. |
| 150 | “functionally-impaired”.mp. |
| 151 | Physical function*.mp. |
| 152 | "Physical frail*".mp. |
| 153 | Debility.mp. |
| 154 | 145 or 146 or 147 or 148 or 149 or 150 or 151 or 152 or 153 |
| 155 | exp Exercise/ |
| 156 | exp Physical Fitness/ |
| 157 | exp Sedentary Behavior/ |
| 158 | exp Rehabilitation/ |
| 159 | exp Exercise Therapy/ |
| 160 | exercis*.mp. |
| 161 | “physical activit*.mp. |
| 162 | physical fitness.mp. |
| 163 | sedentary behavio*r”.mp. |
| 164 | sedentary.mp. |
| 165 | rehabilitat*.mp. |
| 166 | “exercise therapy”.mp. |
| 167 | 155 or 156 or 157 or 158 or 159 or 160 or 161 or 162 or 163 or 164 or 165 or 166 |
| 168 | 144 and 154 and 167 |
| 169 | limit 168 to yr="2000 -Current" |

**Appendix 4. Semi-structured topic guide for scoping review consultation group**

[Introduce yourself]

Thank you for agreeing to talk with me today. The aim of this discussion is to share with you the results of a scoping review [provide further details on aim and methods appropriate to the understanding of the group]

Before we begin, I’d like to remind you that:

- There are no right or wrong answers
- You can choose not to answer any questions you wish, without having to give a reason.
- You can take a break or end the interview at any stage.
- We wont record the meeting, but we will make some notes on what is being said.
- Is there anything you would like to ask me at the moment?

*[share brief presentation outlining the results of the review]*

1. What are your first impressions or reactions to these findings
2. Who is missing from the people who participated

- Long term conditions - mental health?
- Ethnicity
- Age
- Socioeconomic diversity

1. PA and sedentary behaviour has been less explored. Does this surprise you? Do these interventions have a role?
2. What are your thoughts on the barriers/ facilitators to engagement?

- Any that surprise you?
- No work done in sedentary behaviours, do you think barriers and facilitators would be different for these types of interventions?

1. Most delivered at home – thought on this?

- Other delivery methods to explore?

1. How well do you think these interventions would translate into routine NHS care?
2. How do you think sedentary behaviour, physical activity and exercise interventions should be tailored for people living with both frailty and MLTCs?
3. What are your thoughts on how, and how much carers have been involved?

- What else needs to be done in this area?

1. What do you believe the most important gaps in this existing evidence base are?

- Uncertainties
- Areas which are unexplored
- Methodological issues for this field
- Intervention specific issues for this field
- Outcomes which are important but have not been studied

**Closing**

- Thanks
- Anything anyone else would like to add we haven’t mentioned already?
- Anything you thought we would ask that we haven’t?
- Any questions that you would like to ask me?

**Appendix 5. Data extraction items originally outlined in the protocol, and those retained in the refined final data extraction tool.**

|  | Data extraction items outlined within the protocol | Included in the refined data extraction tool |
| --- | --- | --- |
| **Study details** | Author(s) | yes |
|  | Type of publication | yes |
|  | Year of publication | yes |
|  | Country of origin | yes |
| **Description of methodology** | Aims/purpose | yes |
|  | Study design | yes |
|  | Inclusion and exclusion criteria | yes |
|  | Definition of frailty and frailty assessment or proxy functional measure used | yes |
|  | Primary and secondary outcomes | yes |
|  | Where applicable, definition of the carers involved | yes |
|  | Setting/ context (geographical, cultural, social environment and the organisational and political systems in which an intervention occurs) | yes |
|  | Sample size | yes |
|  | Characteristics of the study population, including:  Ethnicity   - Age - Sex - Indicators of socioeconomic status - Presence of cognitive impairment - Number, type and severity of long-term conditions - Level of frailty of participants | yes |
|  | Where applicable, characteristics of carers or family members or significant others, including relationship to care receiver | yes |
|  | Quality Appraisal | no |
| **Description of intervention delivered** | Focus of the intervention (sedentary behaviour, physical activity, exercise or combination) | yes |
|  | If applicable, the type of physical activity/ exercise, including equipment used and an outline of the components included | yes |
|  | The methods used to prescribe the intervention | yes |
|  | The decision rules for determining the starting level | yes |
|  | The intervention duration and dose i.e. the prescribed frequency and intensity, the duration of the intervention and any maintenance period | yes |
|  | The mode of delivery (face to face, virtual, individual or group) | yes |
|  | The decision rules for determining progression | yes |
|  | Details of how the program was progressed and how this was monitored | yes |
|  | The location/ setting of delivery (e.g., home-based or in-centre) including any necessary infrastructure or other relevant features | yes |
|  | Details, methods of, and reasons for tailoring, personalisation, or adaptation. | yes |
|  | Details and methods of any modifications to the intervention during the study, particularly in relation to periods of ill-health and fluctuating symptomology | no |
|  | Intervention rationale, programme theory, or goals | no |
|  | The physical or informational materials used in the intervention, including those provided to participants or used in intervention delivery or in training of intervention providers | no |
|  | The procedures, activities, and/or processes used in the intervention, including any enabling or supportive activities, motivation strategies used (e.g. counselling/education; environmental modification; prompting; self-monitoring; social comparison; financial incentives) | no |
|  | Amount of supervision, including contact time | yes |
|  | The intervention providers, including their qualifications/expertise, background and any training provided to them | yes |
|  | Description of how carers, relatives or significant others are included within the design, development, or delivery of the intervention | yes |
|  | How intervention fidelity was assessed, and by whom, including methods for measuring adherence will also be included | yes |
|  | Strategies used to maintain or improve fidelity | yes |
|  | How well the intervention was delivered as planned, including recorded levels of adherence to the programme | yes |
| **Outcomes** | Outcomes description | yes |
|  | Reported outcome effects- from the paper | yes – taken from the abstract |

**Appendix 6. Outcome measures used within included studies categorised according to the domains of the World Health Organisation International Classification of Functioning and Disability (WHO-ICF).**

| **Outcomes relating to body functions and structures** | | | | | | | | | | | | |
| --- | --- | --- | --- | --- | --- | --- | --- | --- | --- | --- | --- | --- |
| **Symptoms and sensation** | **Fitness and exercise capacity** | **Sleep** | **Cognition** | **Biomarkers** | **Health status** | **Cardiorespiratory function** | **Anthropometrics and body composition** | **Measures related to joint and muscle** | **Disease specific measures** | **Frailty measures** | **Mental health and psychological wellbeing** | **Dialysis measures** |
| Brief pain inventory | 400m walk test | Epworth Daytime Sleepiness Questionnaire | Abbreviated Mental Test Score | Albumin | Health status questionnaire | Blood pressure | Anthropometrics | Sarcopenia (method of measurement not described) | Age, dyspnoea, airflow obstruction (ADO) index | Clinical Frailty Scale (CFS) | Connor-Davidson Resilience Scale (CD-RISC) | Episodes of intradialytic hypotension |
| Dyspnoea 12 questionnaire | Six minute walk test | General sleep disturbance | Attention and psychomotor speed | Alkaline phosphatase | Karnofsky performance scale | Carotid pulse wave velocity | Body mass Index | 30 s arm curl | COPD Assessment Test (CAT) | Edmonton | Outcomes Expectation for Exercise scale | Kt/V (a measure of dialysis adequacy) |
| Functional Assessment of Chronic Illness Therapy – Fatigue Scale (FACIT) | Anaerobic threshold | Insomnia severity index | Mini-Addenbrooke's Cognitive Examination | Blood urea nitrogen | London handicap scale | Forced expiratory volume (FEV1) | Body composition (DEXA and bioelectrical impedance analysis) | Arm lifting test | Hip dysfunction and Osteoarthritis Outcome Score/ Knee injury and Osteoarthritis Outcome Score (HOOS/KOOS) | Evaluative Frailty Index for Physical Activity (EFIP) | Warwick-Edinburgh Mental Wellbeing Scale (WEMWBS) |  |
| Fatigue visual analogue scale (VAS) | Duke Activity Status Index | Pittsburgh Sleep Quality Index | Mini Mental State Examination | Brain-derived neurotrophic factor | Sickness impact profile | he ratio of the forced expiratory volume in the first One second to the forced vital capacity of the lungs (FEV1/FVC) | Bone density | Hand grip strength | Initial claudication distance | FRAIl scale | 3-item Loneliness scale |  |
| London Evaluation of Illness (LEVIL) | Endurance Shuttle Walk Test | Sleep diary | Montreal Cognitive Assessment | Calcidol |  | Forced vital capacity (FVC) | Body weight | Medical Research Council Sum Score | Lequesne algofunctional index | Frailty index | 5 item Pearlin mastery scale |  |
| MD Anderson Symptom Inventory for lung cancer (MDASI-LC) | Incremental Shuttle Walk Test |  | Paced Auditory Serial Addition Test | Calcium |  | Heart rate |  | Number of knee bends per 30 secs | Modified health assessment questionnaire - rheumatology | Fried frailty phenotype | Anxiety and depression scale |  |
| Monofilament sensation testing | Two minute step in place |  | Saint Louis University mental status exam | Creatinine |  | Lung capacity |  | One leg heel raise | Occiput to wall distance | Multidimensional Prognostic Index (MPI) | Beck depression inventory |  |
| MRC dyspnoea score | Two minute walk test |  | Short portable mental status questionnaire (SPMSQ), | C Reactive Protein |  | Left ventricular ejection fraction |  | Pinch test | Oxford hip and knee | SHARE-FI | BREQ-2, Behavioural Regulation in Exercise Questionnaire, version 2; |  |
| Multidimensional fatigue inventory | Minute ventilation/carbon dioxide production (VE/VCO_2_) slope |  | Simple reaction test | D-dimer |  | Mean arterial pressure |  | Muscle power | Parkinson Disease Questionnaire-39 (PDQ-39) | Study of Osteoporotic Fracture (SOF) | Cardiac self-efficacy scale |  |
| Pain VAS | Peak oxygen consumption (VO_2_ peak) |  | Trail making test | Estimated glomerular filtration rate (eGFR) |  | Maximum static expiratory pressure |  | Range of movement | Rutherford claudication distance | Tilburg | Centre for Epidemiologic Studies Depression Scale (CES-D) |  |
| Palliative Care Outcome Scale - renal | Oxygen consumption during the first ventilatory threshold (VO_2_VT1) |  | Verbal fluency | Episodes hypoglycaemia |  | Maximum static inspiratory pressure |  | Strength, Assistance in walking, Rise from a chair, Climb stairs, and Falls (SARC-F) questionnaire | St Georges respiratory questionnaire |  | Coping strategies questionnaire |  |
| Self-reported dizziness |  |  |  | Factor VIII |  | Maximum Voluntary Ventilation test |  | Muscle strength | Timed loaded standing |  | Depression, Anxiety and Stress Scale - 21 Items  (DASS 21) |  |
| Self-reported dyspnoea |  |  |  | Fasting blood glucose |  |  |  |  | Unified Parkinson’s disease rating scale |  | Dialysis patient-perceived Exercise Benefits and Barriers Scale (DPPEBBS) |  |
|  |  |  |  | Fibrinogen |  |  |  |  | Vertebral fracture |  | Exercise self efficacy |  |
|  |  |  |  | Glucose tolerance test |  |  |  |  | Western Ontario and McMaster Universities Arthritis Index (WOMAC) |  | General self-efficacy scale (GSES) |  |
|  |  |  |  | Granulocytes |  |  |  |  |  |  | Hospital Anxiety and Depression Scale (HADS) |  |
|  |  |  |  | Haemoglobin |  |  |  |  |  |  | Health empowerment scale for older |  |
|  |  |  |  | HbA1c |  |  |  |  |  |  | Intensive care psychological assessment tool |  |
|  |  |  |  | haematocrit (HCT) |  |  |  |  |  |  | Kessler Psychological Distress Scale (K10) |  |
|  |  |  |  | High density lipoprotein (HDL) cholesterol |  |  |  |  |  |  | Life satisfaction index |  |
|  |  |  |  | Interleukin 10 (Il-10) |  |  |  |  |  |  | Lille Apathy Rating Scale (LARS) |  |
|  |  |  |  | Interleukin-1 alpha (IL1a) |  |  |  |  |  |  | Measure of actualization of potential (MAP), |  |
|  |  |  |  | IL1 receptor accessory protein (IL1RAcP) |  |  |  |  |  |  | Mental health inventory |  |
|  |  |  |  | Interleukin 6 (Il-6) |  |  |  |  |  |  | Patient Health Questionnaire -8 (PHQ-8) |  |
|  |  |  |  | Insulin |  |  |  |  |  |  | Psychological and behavioural constructs of current exercise behaviour, habit strength, intention, attitude, social influence |  |
|  |  |  |  | Low density lipoprotein (LDL) cholesterol |  |  |  |  |  |  | Rosenberg Self Esteem Scale |  |
|  |  |  |  | Leukocyte count |  |  |  |  |  |  | Self-Rated Abilities for Health Practices questionnaire |  |
|  |  |  |  | Lymphocyte count |  |  |  |  |  |  | Stages of change |  |
|  |  |  |  | Mean corpuscular haemoglobin (MCH) |  |  |  |  |  |  |  |  |
|  |  |  |  | Mean corpuscular haemoglobin concentration (MCHC) |  |  |  |  |  |  |  |  |
|  |  |  |  | Mean corpuscular volume (MCV) |  |  |  |  |  |  |  |  |
|  |  |  |  | Monocyte count |  |  |  |  |  |  |  |  |
|  |  |  |  | Myeloperoxidase (MPO) |  |  |  |  |  |  |  |  |
|  |  |  |  | Mean Platelet Volume (MPV) |  |  |  |  |  |  |  |  |
|  |  |  |  | Parathyroid hormone |  |  |  |  |  |  |  |  |
|  |  |  |  | Phosphate |  |  |  |  |  |  |  |  |
|  |  |  |  | Phosphorous |  |  |  |  |  |  |  |  |
|  |  |  |  | Platelets |  |  |  |  |  |  |  |  |
|  |  |  |  | Potassium |  |  |  |  |  |  |  |  |
|  |  |  |  | Protein Carbonylation |  |  |  |  |  |  |  |  |
|  |  |  |  | Proteins |  |  |  |  |  |  |  |  |
|  |  |  |  | Red blood cell count (RBC) |  |  |  |  |  |  |  |  |
|  |  |  |  | Red cell distribution width (RBCDW) |  |  |  |  |  |  |  |  |
|  |  |  |  | Sodium |  |  |  |  |  |  |  |  |
|  |  |  |  | Thyroid-stimulating hormone, |  |  |  |  |  |  |  |  |
|  |  |  |  | Tumour necrosis factor alpha (TNF-a) |  |  |  |  |  |  |  |  |
|  |  |  |  | TNF-a// il-10 ratio |  |  |  |  |  |  |  |  |
|  |  |  |  | Total cholesterol |  |  |  |  |  |  |  |  |
|  |  |  |  | Transaminases |  |  |  |  |  |  |  |  |
|  |  |  |  | Transferrin |  |  |  |  |  |  |  |  |
|  |  |  |  | Triglycerides |  |  |  |  |  |  |  |  |
|  |  |  |  | Urea |  |  |  |  |  |  |  |  |
|  |  |  |  | Uric acid |  |  |  |  |  |  |  |  |
|  |  |  |  | Vitamin D |  |  |  |  |  |  |  |  |
|  |  |  |  | White blood cell count |  |  |  |  |  |  |  |  |

| **Outcomes relating to activity and participation** | | | | | |
| --- | --- | --- | --- | --- | --- |
| **Balance, mobility and physical function** | **Falls and falls related** | **Physical activity** | **Quality of life** | **Disability and ADLS** | **Social support** |
| Alternate step test (AST) | Falls | Accelerometery | Kings’ college hospital vascular QoL score | Barthel | Duke social support |
| Back scratch test | Falls efficacy scale | Community Healthy Activities Model Program for Seniors questionnaire | Assessment of Quality-of-Life instrument | Frenchay activities index | Medical outcome study social support survey |
| 10m walk test | Fear of falling | Frändin-Grimby scale | Chronic Respiratory Questionnaire (CRQ) | Fried pre-clinical disability screening |  |
| 20m walk test | Survey of Activities and Fear of Falling in the Elderly (SAFFE) | Frequency of outdoor walks | EQ-5D | Functional Autonomy Measurement System (SMAF) |  |
| 4 stage balance test |  | Human activity profile | Functional assessment in cancer scales | Functional independence measure |  |
| 40m walk test |  | International Physical Activity Questionnaire (IPAQ) | Healthy days measure | Functional Status Questionnaire |  |
| 50ft walk test |  | Longitudinal Aging Study Amsterdam Physical Activity Questionnaire (LAPAQ) | Kansas city myopathy | General self-report of ADLS |  |
| 5m walking test |  | Physical Activity Assessment Inventory (PAAI) | Kidney Disease and Quality of Life Instrument (KDQoL-36) | Groningen activity restriction scale |  |
| 6.1-m backward walking test |  | PASE | MacNew questionnaire | Katz index of independence in activities of daily living |  |
| 9 hole peg test |  | Pedometer step count | Mini osteoporosis quality of life questionnaire | The **Lawton** Instrumental Activities of Daily Living Scale (IADL) |  |
| Activities-Specific Balance Confidence Scale (ABC) |  | Physical activity questionnaire of the University of Laval | Peripheral Artery Disease quality of life questionnaire (PAD-QoL) | Summary disability score |  |
| Bedside assessments of basic functional mobility |  | Physical activity recall | Prostate cancer related QoL | Technology Activities of Daily Living Questionnaire (T-ADLQ) |  |
| Berg balance |  | Rapid Assessment of Physical Activity (RAPA) | Patient-Reported Outcomes Measurement Information System (PROMIS) | The Amsterdam linear disability scale |  |
| Balance Outcome Measure for Elder Rehabilitation (BOOMER) |  | Self-report physical activity | Sarcopenia quality of life questionnaire (SARQoL) |  |  |
| Balance Performance-Oriented Mobility Assessment (BPOMA) |  | Stanford 7-day Physical activity recall | Short form 12 health survey (SF-12) |  |  |
| Chair stand (unspecified) |  |  | Short form 36 health survey (SF-36) |  |  |
| Climbing stairs questionnaire (CSQ15) |  |  | Veterans RAND 12 item health survey |  |  |
| Clinical outcomes variable score |  |  |  |  |  |
| De Morton mobility index |  |  |  |  |  |
| Dynamic gait index |  |  |  |  |  |
| Elderly mobility scale |  |  |  |  |  |
| Energy cost of walking |  |  |  |  |  |
| Frailty and Injuries: Cooperative Studies of Intervention Techniques (FICIST- 4) |  |  |  |  |  |
| Forward backward trunk flexion test |  |  |  |  |  |
| Freezing of gait questionnaire |  |  |  |  |  |
| Functional Ambulation Categories |  |  |  |  |  |
| Functional reach |  |  |  |  |  |
| Gait abnormality rating |  |  |  |  |  |
| Gait and balance parameters measured via gait rite and other measurement systems |  |  |  |  |  |
| Gait Efficacy Scale (GES) |  |  |  |  |  |
| Gait speed |  |  |  |  |  |
| Grocery shelving test |  |  |  |  |  |
| Lateral reach |  |  |  |  |  |
| Life space assessment |  |  |  |  |  |
| Late-Life Function & Disability Instrument (LLFDI) |  |  |  |  |  |
| Maximum walking distance |  |  |  |  |  |
| Mini Balance Evaluation Systems Test (Mini-BESTest) |  |  |  |  |  |
| Patient-Specific Functional Scale |  |  |  |  |  |
| Physical function ICU test (PFIT) |  |  |  |  |  |
| Physical Performance Test (PPT) |  |  |  |  |  |
| Rhomberg test |  |  |  |  |  |
| Rising and sitting down questionnaire |  |  |  |  |  |
| Self-report mobility |  |  |  |  |  |
| Seniors fitness test |  |  |  |  |  |
| Sensory organisation test |  |  |  |  |  |
| Sit and reach |  |  |  |  |  |
| Short Physical Performance Battery (SPPB) |  |  |  |  |  |
| Sit to stand in ten seconds (STS10) |  |  |  |  |  |
| Sit to stand three repetitions (STS3) |  |  |  |  |  |
| Sit to stand in thirty seconds (STS30) |  |  |  |  |  |
| Sit to stand five repetitions (STS5) |  |  |  |  |  |
| Sit to stand in sixty seconds (STS60) |  |  |  |  |  |
| Tandem stand |  |  |  |  |  |
| Timed obstacle course |  |  |  |  |  |
| Timed pick up |  |  |  |  |  |
| Timed stair climbing test |  |  |  |  |  |
| Tinetti |  |  |  |  |  |
| Timed up and go (TUAG) |  |  |  |  |  |
| Unilateral stance |  |  |  |  |  |

| **Outcomes relating to environmental factors and other domains** | | | | | |
| --- | --- | --- | --- | --- | --- |
| **Nutrition** | **Feasibility and acceptability** | **surgical/ post op outcomes** | **carer health measures** | **Healthcare utilisation and health economics** | **Other** |
| Controlling nutritional status (CONUT) | Feasibility | Clavien–Dindo complication score | Caregiver strain | Advanced care planning | Death |
| Binge eating | Patient satisfaction, | Comprehensive complication index | Carer EQ-5D | Discharge destination | Number of medications |
| Food frequency questionnaire | Feasibility of Intervention Measure (FIM) | Post-op complications | Carer Hospital anxiety and depression scale (HADS) | Emergency department visits |  |
| Food quality survey for the older adults (ECAAM) | Intervention Appropriateness Measure (IAM) |  | Time spent caring | Healthcare utilisation questionnaire |  |
| Food recall | Satisfaction with intervention |  | Zarit caregiver burden scale | Hospitalisations |  |
| Malnutrition and inflammation score |  |  |  | incremental cost-effectiveness ratio |  |
| Meaning of food questionnaire (MOF-8) |  |  |  | Institutionalisation in residential or nursing home |  |
| Mini Nutritional Assessment – Short Form (MNA-SF) |  |  |  | Length of stay |  |
| Nutrition score |  |  |  | Requirement for a package of care |  |
| SCREEN 14 |  |  |  | Primary care visits |  |
| Short nutritional assessment questionnaire (SNAQ) |  |  |  | Readmissions |  |

*The International Classification of Functioning, Disability and Health (ICF) provides a common framework for the description of health and health-related states. Body Functions are physiological functions of body systems (including psychological functions).Body Structures are anatomical parts of the body such as organs, limbs and their components .Activity is the execution of a task or action by an individual. Participation is involvement in a life situation. Environmental Factors make up the physical, socal and attitudinal environment in which people live and conduct their lives.^46^*

| **Appendix 7. Characteristics of included studies** | | | | | | | | | | | | | | | | | | | | |
| --- | --- | --- | --- | --- | --- | --- | --- | --- | --- | --- | --- | --- | --- | --- | --- | --- | --- | --- | --- | --- |
| **Author [Country]** | | **Study design** | **Frailty or function proxy measure used** | | **Multimorbidity measure** | **Total number of participants recruited** | **Age across all arms** | | | **Intervention summary (all arms)** | **Additional Interventions** | | **Frequency (weekly unless otherwise stated)** | | **Intensity** | **Time (mins)** | | **Duration (weeks)** | **Setting** | **Carer involvement** |
| **Abdelbasset 2022 [Saudi]^50^** | | Parallel RCT | Sit to stand 60 | | Index condition (CKD), with majority of sample living with multiple conditions | 50 | 51 | | | Aerobic exercise (cycling and treadmill) and resistance training | n/a | | 3 | | 70-80% HRmax (aerobic) 50% 1RM (resistance) | 30 | | 12 | Outpatient | No |
| **Ageberg 2013 [Sweden]^51^** | | Controlled before and after study | Sit to stand 5 | | Index condition (OA), with majority of sample living with multiple conditions | 130 | 68 | | | Functional and resistance training | n/a | | 2 | | Not reported | 60 | | 15 | Outpatient | No |
| **Anding 2015 [Germany]^52^** | | Non-randomised experimental study | Timed up and go (TUG) | | Index condition (CKD), with majority of sample living with multiple conditions | 46 | 63 | | | Aerobic exercise (cycling) and resistance training | n/a | | 2 | | Patients started with weights/TheraBand’s inducing a subjectively perceived intensity of "somewhat hard" | 60 | | 60 | Outpatient dialysis | No |
| **Arrieta 2019 [Spain]^53^** | | Parallel RCT | Frailty phenotype (fried); Tilburg Frailty SOF | | Older population, with majority of sample living with multiple conditions | 112 | 85 | | | Resistance training, balance training | n/a | | 2 | | "Moderate intensity" | 60 | | 26 | Nursing or residential home | No |
| **Bean 2004 [United States]^54^** | | Parallel RCT | Sit to stand 5 | | Older population, with majority of sample living with multiple conditions | 21 | 78 | | | Resistance training using a weighted vest | n/a | | 3 | | RPE | 30 | | 12 | Home and community | No |
| **Bennell 2020 [Australia]^55^** | | Parallel RCT | Sit to stand 30 | | Inclusion criteria of knee OA and obesity | 128 | 62 | | | Resistance exercise (compared weightbearing and non-weightbearing) | n/a | | 2 | | 5 and 8 out of 10 (hard to very hard) on the modified Borg Rating of Perceived Exertion category-ratio-10 (CR-10) scale for strength training | 40 | | 12 | Outpatient and home | No |
| **Bennett 2018 [United States]^56^** | | Parallel RCT | Short physical performance battery; Gait speed | | Older population, with majority of sample living with multiple conditions | 23 | 74 | | | Aerobic exercise (line dancing) | n/a | | 2 | | Borg rating of less than 15 | 60 | | 8 | Community | No |
| **Bennett 2020 [United States]^57^** | | Parallel RCT | Timed up and go (TUG); Gait speed; Sit to stand 30; Sit to stand 60 | | Index condition (CKD), with majority of sample living with multiple conditions | 36 | 58 | | | Aerobic (cycling or walking) and resistance exercise | n/a | | 3 | | "Moderate intensity" | 30 | | 12 | Outpatient and home | No |
| **Berkel 2022 [Netherlands]^58^** | | Parallel RCT | Timed up and go (TUG) | | Index condition (solid organ cancer), with Charlson co-morbidity index indicating multimorbidity | 74 | 73 | | | Aerobic (cycling) and resistance training | n/a | | 3 | | "Moderate to high " | 60 | | 3 | Outpatient | No |
| **Bernabei 2022 [Multi-country]^59^** | | Parallel RCT | Short physical performance battery | | Older population, with majority of sample living with multiple conditions | 1519 | 78 | | | Aerobic exercise (walking and cycling), balance and resistance training | Nutrition counselling | | 3 | | Participants were asked to walk at an intensity of 13 (somewhat hard). Lower extremity strengthening exercises were performed at an intensity of 15 or 16 (hard). | 30 | | 156 | Outpatient and home | No |
| **Bohm 2014 [Canada]^60^** | | Parallel RCT | Sit to stand 30 | | Index condition (CKD), with majority of sample living with multiple conditions | 60 | 52 | | | Aerobic exercise (cycling) compared with a pedometer group | n/a | | 3 | | The goal intensity for each session was 12-14 (moderate to somewhat hard) on the 20-point Borg RPE. | 60 | | 24 | Outpatient dialysis | No |
| **Bouaziz 2018 [France]^61^** | | Parallel RCT | Timed up and go (TUG) | | Older population, with Charlson co-morbidity index indicating multimorbidity | 60 | 74 | | | Aerobic exercise (cycling) | n/a | | 2 | | Each session involved six 5 minutes of exercise combining 4-minute cycling at the preintervention VT1 workload and 1-minute cycling at 40% of the preintervention VT1 workload | 30 | | 9.5 | Community | No |
| **Brovold 2013 [Norway]^62^** | | Parallel RCT | Sit to stand 30 | | Older population, with majority of sample living with multiple conditions | 115 | 78 | | | aerobic (walking) resistance exercise and balance training | n/a | | 3 | | Moderate intensity was defined to be between 11 and 13 and high intensity between 15 and 17 on the Borg Scale | 60 | | 12 | Post discharge | No |
| **Brown 2006 [United States]^63^** | | Mixed methods feasibility study | Timed up and go (TUG) | | Older population, with Charlson co-morbidity index indicating multimorbidity | 10 | 70 | | | Resistance, functional and mobility training | n/a | | 7 | | Not reported | 30 | | 12 | Post discharge | No |
| **Budui 2019 [Italy]^64^** | | Pre/post design | Sit to stand 30 | | Index condition (Obesity), with majority of sample living with multiple conditions | 259 | 59 | | | Aerobic exercise (cycling or treadmill) and resistance training | Nutrition and psychological support | | 7 | | Aerobic intensity was set around VT1 | 120 | | 3 | Hospital inpatient | No |
| **Busch 2012 [Germany]^65^** | | Parallel RCT | Timed up and go (TUG) | | Index condition (CVD), with majority of sample living with multiple conditions | 121 | 78 | | | Cardiac rehabilitation and additional resistance and balance training | n/a | | 3 | | 60% of one repetition maximum. The difficulty of balance tasks was individually increased. Resistance and balance training were controlled using a rating of perceived exertion of 13 (somewhat hard) on a scale from 6 to 20. | 120 | | 3 | Outpatient rehab | No |
| **Caldo-Silva 2023 [Portugal]^67^ᶲ** | | Non-randomised experimental study | Frailty phenotype (fried) | | Older population, with Charlson co-morbidity index indicating multimorbidity | 35 | 83 | | | Walking, resistance, and balance training | Nutritional supplementation | | 2 | | RPE 4-6 out of 10 | Not reported | | 16 | Nursing or residential home | No |
| **Cameron 2013 [Australia]^68^ᶳ** | | Parallel RCT | Frailty phenotype (fried) | | Older population, with majority of sample living with multiple conditions | 241 | 83 | | | Aerobic (not specified), resistance and balance training | Nutrition supplementation, psychology, case management, social engagement | | 5 | | Not reported | Not reported | | 52 | Home | No |
| **Campo et al 2020 [Italy]^69^** | | Parallel RCT | Frailty phenotype (fried) | | Index condition (CVD), with majority of sample living with multiple conditions | 235 | 76 | | | Aerobic (treadmill) strength and balance | n/a | | 3 | | "Moderate intensity" | 40 | | 52 | Outpatient | The exercise programme was extensively described to...family members.  Family members were free to contact... In case of doubts or request of additional information and/or clarification. |
| **Carballeira 2021 [Spain]^70^** | | Parallel RCT | Balance Performance Orientated Mobility Assessment; Short physical performance battery | | MLTC population | 24 | 81 | | | Aerobic (cycling) exercise | n/a | | 3 | | In the first training  phase (i.e., the first three weeks), participants were requested to cycle simultaneously with the upper and lower  limbs at an intensity equivalent to a perception of 3 (i.e., easy to somewhat moderate) on the OMNI-RPE.  Subsequently, in the second training phase (i.e., the last three weeks), they were requested to cycle at an intensity  equivalent to an OMNI-RPE score of 6 (i.e., somewhat hard). | 20 | | 6 | Outpatient | No |
| **Carli 2020 [Canada]^71^** | | Parallel RCT | Frailty phenotype (fried) | | MLTC population | 120 | 79 | | | Aerobic (recumbent stepper) exercise and resistance training | Nutrition and psychological support | | 1 | | “Moderate intensity" | 60 | | 4 | Prehabilitation and post op rehab | No |
| **Cederbom 2019 [Norway]^72^** | | Parallel RCT | Short physical performance battery | | Older population, with majority of sample living with multiple conditions | 105 | 85 | | | Functional training | Behaviour change support | | 1 | | Not reported | Not reported | | 12 | Home | No |
| **Celli 2022 [United States]^73^** | | Parallel RCT | Modified physical performance test | | Index condition (T2D), with majority of sample living with multiple conditions | 100 | 72 | | | Aerobic exercise (treadmill, cycling or stair climbing), resistance training | Nutrition support | | 3 | | Participants exercised at 65% of their peak heart rate, which was gradually increased to 70-85%. | 90 | | 52 | Outpatient | No |
| **Cesari 2015 [United States]^74^** | | Parallel RCT | Frailty phenotype (fried) | | Older population, with majority of sample living with multiple conditions | 424 | 77 | | | Aerobic (walking, cycling) exercise, resistance, and balance training, stretching and flexibility | n/a | | 3 | | Walking RPE 13. Lower extremity strengthening exercises were performed at an intensity of 15-16 on the Borg scale. | 60 | | 52 | Outpatient | No |
| **Chan 2017 [Taiwan]^75^** | | Parallel RCT | Timed up and go (TUG) | | Older population, with majority of sample living with multiple conditions | 289 randomised | 72 | | | Aerobic (walking), resistance and balance training | Psychotherapy and education | | 2 | | Not reported | 60 | | 28 | Hospital inpatient | No |
| **Chen 2010 [United States]^76^** | | Pilot RCT | Short physical performance battery | | Index condition (CKD), with majority of sample living with multiple conditions | 50 randomised | 69 | | | Resistance training | n/a | | 2 | | Rate of perceived exertion (RPE) modified OMNI Scale [18], with a target moderate intensity of 6 (somewhat hard) out of 10 (extremely hard), equivalent to 60% of a one-repetition maximum | Not reported | | 24 | Outpatient dialysis | No |
| **Chen 2016 [Taiwan]^77^** | | Cluster RCT | Handgrip | | Older population, with majority of sample living with multiple conditions | 127 | 79 | | | Resistance training | n/a | | 3 | | Not reported | 40 | | 52 | Nursing or residential home | No |
| **Chen 2020 [China]^78^** | | Parallel RCT | Timed up and go (TUG); Sit to stand 30 | | MLTC population | 70 | 65 | | | Resistance training | n/a | | 3 | | RPE of 13, with the scale ranging from 6 to 20 | Not reported | | 12 | Home | Family members shown exercises to ensure they were performed correctly |
| **Chiu 2018 [Taiwan]^79^** | | Non-randomised experimental study | Handgrip | | Index condition (Obesity), with CIRS indicating multimorbidity | 123 | 80 | | | Resistance training | n/a | | 2 | | assessed with RPE | 60 | | 9 | Nursing or residential home | No |
| **Clegg 2014 [UK]^80^** | | Pilot RCT | Edmonton Frail Scale | | Older population, with Charlson co-morbidity index indicating multimorbidity | 84 | 79 | | | Mobility and transfers, resistance, and balance training | n/a | | 5 | | Not reported | 15 | | 12 | Home | No |
| **Comans 2010 [Australia]^81^** | | Parallel RCT | Timed up and go (TUG) | | Older population, with majority of sample living with multiple conditions | 107 | 79 | | | Aerobic (walking) exercise, resistance and balance training, functional training, tai chi, physical activity (walking and ADLS) | Education and home safety | | 1 | | Not reported | 10 minutes for home balance exercise, others not reported | | 8 | Community and outpatient rehab | No |
| **da Silva 2015 [Brazil]^82^** | | Parallel RCT | Timed up and go (TUG); Sit to stand 30 | | Index condition (OA), with majority of sample living with multiple conditions | 41 | 58 | | | Aerobic (cycling), resistance and balance training, mobility, and transfers | Education | | 2 | | Moderate level of perceived effort according to RPE | 60 | | 8 | Outpatient | No |
| **de Rooji 2017[Netherlands]^83^** | | Parallel RCT | EFIP | | Inclusion criteria of OA with additional co-morbidity | 126 | 63 | | | Aerobic (not specified) exercise, resistance and balance training, functional training, stretching and flexibility | n/a | | 2 | | 50-80% of VO2max/HRR/HRmax, maximal workload (moderate intensity) 30-<40% or VO2max/HRR/HRmax maximal workload (light intensity)   40-60% of 1-RM endurance resistance training  60-80 % of 1RM power training | 30-60 | | 20 | Outpatient | No |
| **Deer 2019 [United States]^84^** | | Parallel RCT | Short physical performance battery; Gait speed | | Older population, with Charlson co-morbidity index indicating multimorbidity | 113 | 79 | | | Resistance training | Nutritional supplementation | | 3 | | Not reported | Not reported | | 4 | Post discharge | No |
| **De Liao 2017 [Taiwan]^85^** | | Parallel RCT | Timed up and go (TUG); Sit to stand 30; Handgrip | | Index condition (Obesity), with CIRS indicating multimorbidity | 46 | 67 | | | Resistance training | n/a | | 3 | | RPE scale | 60 | | 12 | Outpatient rehab | No |
| **deVries 2016 [Netherlands]^86^** | | Parallel RCT | EFIP | | Older population, with CIRS indicating multimorbidity | 130 | 78 | | | Aerobic (not specified) exercise, resistance, balance, functional training | Behaviour change support | | Not reported | | Not reported | 90 intake session, 30 intervention session | | 24 | Outpatient | Active involvement in reaching these goals in which family, friends, or (informal) caregivers are involved.  Intervention includes a step on meaningful activities at home with help from family, friends, and/or professionals |
| **Dimori 2017 [Italy]^87^** | | Pre/post design | Short physical performance battery; Gait speed; Handgrip | | Older population, with CIRS indicating multimorbidity | 39 | 86 | | | Aerobic (not specified) exercise and resistance training | Nutrition support | | 3 | | Not reported | 40 | | 52 | Nursing or residential home | No |
| **Donesky 2017 [United States]^88^** | | Non-randomised experimental study | Sit to stand 30 | | Inclusion criteria of HF and COPD | 15 | 72 | | | Yoga | n/a | | 2 | | Not reported | 55 | | 8 | Telemedicine | No |
| **Evistigneeva 2016 [Russia]^90^** | | Parallel RCT | Timed up and go (TUG) | | Index condition (Osteoporosis), with majority of sample living with multiple conditions | 78 | 69 | | | Resistance training | n/a | | 2 | | Not reported | 40 | | 52 | Outpatient | No |
| **Faes 2011 [Netherlands]^91^** | | Parallel RCT | Frailty phenotype (fried) | | Older population, with CIRS indicating multimorbidity | 36 pairs of patients and caregivers | 78 | | | Resistance training, functional training, mobility, and transfers | Psychological support | | 2 | | Not reported | 120 | |  | Outpatient | Included in the intervention in a dyad with patient. |
| **Ferreira 2018 [Brazil]^92^** | | Parallel RCT | Frailty phenotype (fried) | | Older population, with majority of sample living with multiple conditions | 45 | 75 | | | Aerobic (not specified) exercise, resistance training, mobility, and transfers, stretching and flexibility | n/a | | 3 | | RPE 5 and 7 of RPE on an adapted Omni scale | 40 | | 12 | Nursing or residential home | No |
| **Fielding 2017 [United States]^93^** | | Parallel RCT | Short physical performance battery | | Older population, with majority of sample living with multiple conditions | 1635 | 78 | | | Aerobic (walking) exercise, resistance and balance training, functional training, physical activity (walking) | n/a | | 2 | | RPE 13 for walking. Lower extremity strengthening exercises are performed at an intensity of 15-16 | 60 | | 130 | Outpatient and home | No |
| **Finamore 2021 [Italy]^94^** | | A secondary analysis of a trial aimed at evaluating  whether resistance training in addition to endurance training  might improve respiratory outcomes. | Timed up and go (TUG) | | Index condition (COPD), with majority of sample living with multiple conditions | 64 | 74 | | | Aerobic (not specified) exercise, resistance exercise | pharmacotherapy optimisation | | 3 | | Activation phase mild intensity (resistance training at 40% of 1-RM). Second intervention phase: endurance group at moderate intensity according to Borg scale values and endurance and resistance group 0-50% of heart rate reserve and the resistance training at 50% of 1-RM | 45 | | 16 | Outpatient rehab | No |
| **Fisher 2018 [Canada]^95^** | | Parallel RCT | Modified physical performance test; Sit to stand 30 | | Older population, with majority of sample living with multiple conditions | 172 | 60 | | | Aerobic (not specified) exercise, resistance training, stretching and flexibility | Education | | 3 | | Moderate intensity | 60 | | 52 | Community and home | No |
| **Fransen 2007 [Australia]^96^** | | Parallel RCT | Timed up and go (TUG) | | Index condition (OA), with self-administered comorbidity questionnaire indicating multimorbidity | 152 | 70 | | | Tai chi and hydrotherapy | n/a | | 2 | | Not reported | 60 | | 12 | Outpatient | No |
| **Gibbs 2020 [Multi-country]^97^** | | Parallel RCT | Sit to stand 5 | | Index condition (Osteoporosis), with majority of sample living with multiple conditions | 141 | 77 | | | Resistance and balance training | Behaviour change support | | 3 | | Not reported | 10-30 aerobic, others not reported | | 32 | Home | No |
| **Gill 2004 [United States]^99^ᶺ** | | Parallel RCT | Gait speed and STS5 | | Older population, with majority of sample living with multiple conditions | 188 | 83 | | | Resistance and balance training | Home hazard assessment and interventions | | 3 | | Not reported | Not reported | | 24 | Home | No |
| **Greenwood 2021 [UK]^100^** | | Parallel RCT | Sit to stand 60 | | Index condition (CKD), with majority of sample living with multiple conditions | 335 | 57 | | | Aerobic (cycling) exercise and resistance training | n/a | | 3 | | Exercise prescription was set at a workload corresponding to 40-75% of VO2 reserve. | 40 | | 24 | Outpatient intradialytic | No |
| **Haider 2017a [Austria]^101^ᶯ** | | Parallel RCT | SHARE-FI | | Older population, with Charlson co-morbidity index indicating multimorbidity | 80 | 82.7 | | | Resistance and balance training, functional training, mobility, and balance | Nutrition education | | 3 | | Not reported | Not reported | | 12 | Not reported | No |
| **Haider 2017b [Austria]^102^ᶯ** | | Parallel RCT | SHARE-FI | | Older population, with Charlson co-morbidity index indicating multimorbidity | 80 | 82 | | | Resistance and balance training, functional training, mobility, and balance | Nutrition education | | 3 | | Not reported | Not reported | | 12 | Not reported | No |
| **Hartley 2021 [UK]^103^** | | Mixed methods feasibility study | Clinical Frailty Scale; SHARE-FI, CFS | | Older population, with Charlson co-morbidity index indicating multimorbidity | 15 | 86 | | | Mobility and transfers | n/a | | twice daily* | | Not reported | Not reported | | 1 | Hospital inpatient | No |
| **Helbostad 2004 [Norway]^104^ᶹ** | | Parallel RCT | Timed up and go (TUG) | | Older population, with majority of sample living with multiple conditions | 77 | 81 | | | Resistance and balance training | n/a | | 2 | | Not reported | Not reported | | 12 | Home and outpatient rehab | No |
| **Helbostad 2004 [Norway]^105^ᶹ** | | Parallel RCT | Timed up and go (TUG) | | Older population, with majority of sample living with multiple conditions | 77 | 81 | | | Resistance and balance training | n/a | | 2 | | Not reported | Not reported | | 12 | Home and outpatient rehab | No |
| **Henwood 2019 [Australia]^106^** | | Mixed methods pre/post design | Short physical performance battery | | Older population, with majority of sample living with multiple conditions | 50 | 82 | | | Resistance and balance training | n/a | | 7 | | Not reported | 10 | | 18 | Home | No |
| **Hewitt 2018 [Australia]^107^** | | Cluster RCT | Short physical performance battery | | Older population, with majority of sample living with multiple conditions | 221 | 86 | | | Resistance and balance training | n/a | | 2 | | 12 to 14 Borg Scale of Perceived Exertion. | Not reported | | 25 | Nursing or residential home | No |
| **Hinrichs 2016 [Germany]^108^** | | Parallel RCT | Timed up and go (TUG); Sit to stand 5; Handgrip | | Older population, with majority of sample living with multiple conditions | 209 | 80 | | | Resistance and balance training and habitual physical activity (walking) | n/a | | 2 | | Moderate (5 or 6 on a 10-point scale) for the strength exercises and walking | 30 minutes outdoor walking, other components not reported | | 12 | Home | No |
| **Hsu 2021 [Taiwan]^109^** | | Parallel RCT | Timed up and go (TUG) | | Inclusion criteria of OA and obesity | 66 | 65 | | | Resistance training | n/a | | 3 | | RPE 13 | Not reported | | 12 | Home telemedicine and outpatient | No |
| **Ilranzo 2018 [Spain]^110^** | | Parallel RCT | Handgrip | | Older population, with majority of sample living with multiple conditions | 81 | 84 | | | Resistance training | Inspiratory muscle training | | 3 | | 40- 60% of maximal isometric muscles  strength (Kg) | 40 | | 12 | Nursing or residential home | No |
| **Jang 2018 [South Korea]^111^** | | A designed-delay study wherein the intervention was rolled out sequentially in three geographic  regions with a planned 6-month interval | Short physical performance battery | | Older population, with majority of sample living with multiple conditions | 187 | 77 | | | Aerobic (step ups and dancing) resistance and balance training | Nutritional supplementation, depression management,  deprescription of medications, and home hazard reduction | | 2 | | Not reported | 60 | | 24 | Community and home | No |
| **Jepma 2021 [Netherlands]ᶭ** | | Parallel RCT | Dutch safety management system | | Index condition (CVD), with Charlson co-morbidity index indicating multimorbidity | 306 | 82 | | | Cardiac rehabilitation | Case management and disease management | | 2 | | Not reported | Not reported | | Not reported | Post discharge | Informal caregivers were involved in the intervention if they were present |
| **Jones 2006 [Australia]^112^** | | Parallel RCT | Timed up and go (TUG) | | Older population, with Charlson co-morbidity index indicating multimorbidity | 160 | 82 | | | Aerobic (walking) exercise, resistance and balance training, functional training | n/a | | twice daily* | | Not reported | Not reported | | 6 | Hospital inpatient | No |
| **Kelly 2016 [United States]^113^** | | Parallel RCT | Timed up and go (TUG); | | Index condition (OA), with majority of sample living with multiple conditions | 38 | 71 | | | Aerobic (walking) exercise, resistance training, mobility, and transfers, stretching and flexibility | n/a | | 2 | | Open-chain exercises were performed at 50% of 1 RM and then 80% of 1 RM | Not reported | | 7 | Outpatient | No |
| **Kerse 2008 [New Zealand]^114^** | | Cluster RCT | Timed up and go (TUG) | | Older population, with majority of sample living with multiple conditions | 682 | 84 | | | Functional training | n/a | | At least once daily* | | Not reported | Not reported | | 24 | Nursing or residential home | No |
| **Kho 2019 [Canada]^115^** | | Pilot RCT | Clinical Frailty Scale | | ICU population with majority of sample living with multiple conditions | 66 | 62 | | | Aerobic (cycling) exercise | n/a | | 5 | | Not reported | 30 | | Duration of the ICU stay | ICU | No |
| **King 2015 [United States]^116^** | | Parallel RCT | Timed up and go (TUG) | | Index condition (PD), with CIRS indicating multimorbidity | 59 | 64 | | | Tai chi, Pilates, and resistance training | n/a | | 3 | | Rate of Perceived Exertion (RPE) | 60 | | 4 | Outpatient and home | No |
| **Kitzman 2021 [United States]^117^** | | Parallel RCT | Frailty phenotype (fried) | | Index condition (HF), with majority of sample living with multiple conditions | 349 | 73 | | | Aerobic (walking) exercise, balance and resistance training, mobility, and transfers | n/a | | 3 | | During the first 2 weeks, target intensity is low (RPE 12) and gradually increased to 13 for endurance training and 15-16 for strength | 60 | | 24 | Post discharge | No |
| **Kocic 2018 [Serbia]^118^** | | Parallel RCT | Timed up and go (TUG) | | Older population, with majority of sample living with multiple conditions | 77 | 78 | | | Resistance and balance training | n/a | | 3 | | Not reported | 40 | | 24 | Nursing or residential home | No |
| **Lauzẻ 2017 [Canada]^119^** | | Parallel RCT | Study Osteoporotic Fractures (SOF) Index | | Older population, with majority of sample living with multiple conditions | 42 | 82 | | | Resistance and balance training | n/a | | 2 | | Light to moderate intensity | 45 | | 12 | Telemedicine | No |
| **Li 2005 [United States]^120^** | | Parallel RCT | Timed up and go (TUG) | | Older population, with majority of sample living with multiple conditions | 256 | 77 | | | Tai chi | n/a | | 3 | | Not reported | 60 | | 26 | Not reported | No |
| **Li 2017 [Taiwan]^121^** | | Non-randomised experimental study | Frailty phenotype (fried) | | Older population, with majority of sample living with multiple conditions | 404 | 78 | | | Resistance and balance training | Nutritional support | | Not reported | | Not reported | Not reported | | 52 | Not reported | No |
| **Liao 2020 [Taiwan]^122^** | | Parallel RCT | Timed up and go (TUG); Gait speed | | Index condition (OA), with CIRS indicating multimorbidity | 60 | 71 | | | Resistance training | n/a | | 2 | | Not reported | 60 | | 12 | Outpatient rehab and home | No |
| **Liu 2015 [Taiwan]^123^** | | Non-randomised experimental study | Sit to stand 60 | | Index condition (CKD), with majority of sample living with multiple conditions | 24 | 39 | | | Aerobic (cycling) exercise | n/a | | 3 | | RPE between 11 (fairly light) and 13 (somewhat hard) | 30 | | 12 | Outpatient intradialytic | No |
| **Liu 2017 [Hong Kong]^124^** | | Non-randomised experimental study | Frailty phenotype (fried) | | Older population, with Charlson co-morbidity index indicating multimorbidity | 85 | 79 | | | Aerobic (circuit based) exercise, resistance, and balance training | Behaviour change support | | 1 | | Moderate intensity via RPE | 60 | | 16 | Outpatient | No |
| **Lo 2021 [Taiwan]^125^** | | Parallel RCT | Sit to stand 30 | | MLTC population | 43 | 64.4 | | | Aerobic (cycling) exercise and habitual physical activity (walking) | Behaviour change support | | 3 | | Aerobic intensity (gradually increased from 50% heart rate reserve to 80% heart rate reserve according to the Karvonen method | 30-50 | | 12 | Outpatient | No |
| **Lorenz 2020 [United States]^126^** | | Pre/post design | Frailty phenotype (fried) | | Index condition (CKD), with majority of sample living with multiple conditions | 27 | 61 | | | Aerobic (treadmill or hand ergometer) exercise, resistance, and balance training, stretching and flexibility | n/a | | 2 | | Moderate intensity | 60 | | 8 | Outpatient rehab | No |
| **Losa-Reyna [Spain]^127^** | | Non-randomised experimental study | Frailty phenotype (fried) | | Older population, with majority of sample living with multiple conditions | 30 | 84 | | | Resistance and balance training | n/a | | 2 | | 30-60% F0 | 45 | | 6 | Not reported | No |
| **Loyola 2018 [Brazil]^128^** | | Pre/post design | Timed up and go (TUG) | | Older population, with Charlson co-morbidity index indicating multimorbidity | 200 | 72 | | | Aerobic (circuit based) exercise, resistance, and balance training | n/a | | 2 | | Based on the max number of repetitions for each exercise | 35-45 | | 12 | Not reported | No |
| **Marti­nez-Velilla 2019 [Spain]^129^** | | Parallel RCT | Short physical performance battery | | Older population, with CIRS indicating multimorbidity | 370 | 87 | | | Resistance and balance training, functional training | n/a | | 7* | | 30% to 60% of the 1-repetition maximum. | 20 | | For the duration of the hospitalisation | Hospital inpatient | Participants and their family members were familiarized with the training procedures before the start of the intervention. |
| **Martel 2018 [Canada]^130^** | | Parallel RCT | SOF | | Older population, with majority of sample living with multiple conditions | 48 | 73 | | | Aerobic (circuit based) exercise, balance, and resistance training | n/a | | 2 | | Not reported | 55 | | 12 | Telemedicine and community | No |
| **Martin-Alemany 2020 [Mexico]^131^** | | Parallel RCT | Sit to stand 5; Handgrip | | Index condition (CKD), with majority of sample living with multiple conditions | 45 | 29 | | | Aerobic (cycling) exercise, resistance training | Nutritional supplementation | | 3 | | n/a | n/a | | 12 | Outpatient intradialytic | No |
| **Maynard 2019 [Brazil]^132^** | | Parallel RCT | Timed up and go (TUG) | | Index condition (CKD), with majority of sample living with multiple conditions | 45 | 46 | | | Resistance and balance training | n/a | | 3 | | RPE 12-14 | 30-60 | | 12 | Outpatient intradialytic | No |
| **Meyer 2022 [Germany]^133^** | | Parallel RCT | Multidimensional Prognostic Index (MPI) | | MLTC population | 110 | 78* | | | Resistance training | Education and structured support | | Not reported | | Not reported | Not reported | | Not reported | inpatient and outpatient | Offered lifestyle counselling to patients and caregivers on admission to the ward. In addition, the team was available for...caregivers...throughout the intervention period if needed. |
| **Morey 2006 [United States]^134^** | | Parallel RCT | Timed up and go (TUG); Sit to stand 30 | | Older population, with majority of sample living with multiple conditions | 175 | 70 | | | Habitual physical activity (walking) | n/a | | 5 | | Moderate intensity | 30 | | 24 | Home | No |
| **Nagai 2018 [Japan]^135^** | | Parallel RCT | Frailty phenotype (fried) | | Older population, with majority of sample living with multiple conditions | 41 | 81 | | | Resistance training, habitual physical activity (walking) | n/a | | 2 | | 50% to 80% of 1 RM, | Not reported | | 24 | Outpatient | No |
| **Nitz 2004 [Australia]^136^** | | Parallel RCT | Timed up and go (TUG) | | Older population, with majority of sample living with multiple conditions | 73 | 76 | | | Aerobic (circuit based) exercise and balance training | n/a | | 1 | | Not reported | 60 | | 10 | Outpatient | No |
| **Nixon 2021 [UK]^137^** | | Mixed methods pilot RCT | Clinical Frailty Scale (CFS) | | Index condition (CKD), with Charlson co-morbidity index indicating multimorbidity | 29 | 78 | | | Aerobic (not specified) exercise, resistance, and balance training | n/a | | 3 | | Moderate intensity | 30-45 | | 12 | Home | No |
| **Oh 2020 [South Korea]^138^** | | Parallel RCT | Study of Osteoporotic Fracture (SOF) Index | | Index condition (OA), with majority of sample living with multiple conditions | 60 | 72 | | | Resistance training | Education | | 3 | | Not reported | Not reported | | 20 | Home | No |
| **Ortega-Pérez et al 2020 [Spain]^139^** | | Pilot RCT | Sit to stand 60; Handgrip | | Index condition (CKD), with Charlson co-morbidity index indicating multimorbidity | 46 | 61 | | | Aerobic (cycling) exercise, resistance, and physical activity (walking) | n/a | | 3 | | RPE 12-15, the load for resistance training was adapted according to 10 rep max | 30 aerobic, no time given for strength | | 16 | Outpatient intradialytic and home | No |
| **Ory 2015 [United States] ^201^** | | Pre/post design | Timed up and go (TUG) | | Older population, with majority of sample living with multiple conditions | 220 | 75 | | | Aerobic (circuit based) exercise, resistance, and balance training, stretching and flexibility | Education | | 2 | | Not reported | 90 | | 12 | Telemedicine | No |
| **O'shea 2007 [Australia]^140^** | | Parallel RCT | Timed up and go (TUG) | | Index condition (COPD), with majority of sample living with multiple conditions | 54 | 68 | | | Resistance training | n/a | | 3 | | Not reported | Not reported | | 12 | Home | No |
| **Papaioannou 2003 [Canada]^141^** | | Parallel RCT | Timed up and go (TUG) | | Index condition (Osteoporosis), with majority of sample living with multiple conditions | 74 | 72 | | | Aerobic (walking) exercise and resistance training, stretching and flexibility | n/a | | 3 | | Not reported | 60 | | 24 | Home | No |
| **Pariser 2013 [United States]^142^** | | Pre/post design | Modified physical performance test | | Index condition (T2D), with majority of sample living with multiple conditions | 57 | 62 | | | Aerobic (seated aerobics) and resistance training | Education | | 4 | | RPE 11 out of 20 (light) and 13 out of 20 (somewhat hard). | 90 | | 10 | Community and home | No |
| **Patil 2015 [Finland]^143^** | | Parallel RCT | Timed up and go (TUG); Sit to stand 5 | | Older population, with CIRS indicating multimorbidity | (n = 409) | 74 | | | Resistance and balance training, functional training | n/a | | 2 | | RPE 14 to 18 (12 when starting) | 5-15 for the home programme 60 for the gym session | | 104 | Community | No |
| **Pizarro-Mena 2022 [Chile]^144^** | | Pre/post design | Tilburg Frailty Indicator; FRAIL scale | | MLTC population | 52 | 71 | | | Aerobic (not specified) exercise, resistance, and balance training | Education | | 3 | | 50-70% HR max for aerobic  60-80% RM for strength | 60 | | 12 | Community | No |
| **Reeves 2017 [United States]^145^** | | Pilot RCT | Frailty phenotype (fried) | | Index condition (HF), with majority of sample living with multiple conditions | 27 | 72 | | | Aerobic (walking) exercise, resistance and balance training, mobility, and transfers | n/a | | 3 | | RPE 12; after 2 weeks increased to 13 for endurance training and 15-16 for strength training | 60 outpatients 30 hospital when feasible home: up to 30 | | 12 | Post discharge | The demands of study participation were discussed in detail with potential participants and, whenever possible, family and/or caregivers. A standardized assessment tool was used to query...degree of support from family members, caregivers. Those...who had a lack of support were considered high risk  for non-adherence and were Not randomized and were excluded from the study. Flexible scheduling, ongoing engagement of the participant's family promoted study adherence and retention. |
| **Rhee 2019 [South Korea]^146^** | | Pre/post design | Sit to stand 60 | | Index condition (CKD), with majority of sample living with multiple conditions | 22 | 57 | | | Aerobic (cycling) exercise and resistance training | n/a | | 3 | | Started at Borg RPE 11, 11% to 60% effort aiming  for Borg RPE 13 to 14, 13% to 75% effort. | 30 | | 24 | Outpatient intradialytic | No |
| **Rodriguez-Manas 2019 [Multi-country]^147^** | | Cluster RCT | Frailty phenotype (fried) | | Index condition (T2D), with majority of sample living with multiple conditions | 964 | 78 | | | Resistance training | Nutrition education | | 2 | | 40-80% of the estimated one-repetition maximum (1RM). | 45 | | 18 | Not reported | No |
| **Rogers 2012 [United States]^148^** | | Parallel RCT | Timed up and go (TUG) | | Older population, with majority of sample living with multiple conditions | 67 | 75 | | | Tai chi | n/a | | 5 | | Not reported | 60 class, then up to 30 at home | | 12 | Not reported | No |
| **Rosko 2021 [United States]^149^** | | Pre/post design | Short physical performance battery | | Index condition (haematological cancer), with The Older Americans Resources and Services (OARS) Questionnaire Physical indicating multimorbidity | 30 | 74 | | | Resistance and balance training, mobility, and transfers | n/a | | 2 | | Not reported | Not reported | | 16 | Community and home | No |
| **Salisbury 2022 [United States]^150^** | | Pilot RCT | Timed up and go (TUG); Sit to stand 5 | | Index condition (PAD), with majority of sample living with multiple conditions | 19 | 75 | | | Aerobic (treadmill vs recumbent stepping) exercise | n/a | | 3 | | Not reported | 60 | | 12 | Outpatient rehab | No |
| **Sanchez-Tocino 2022 [Spain]^151^** | | Non-randomised experimental study | Frailty phenotype (fried) | | Index condition (CKD), with Charlson co-morbidity index indicating multimorbidity | 60 | 82 | | | Aerobic exercise (cycling) and resistance training | n/a | | 3 | | RPE 12-14 | 60 | | 12 | Outpatient dialysis | No |
| **Schwenk 2014 [United States]^152^** | | Pilot RCT | Timed up and go (TUG); Gait speed | | Older population, with majority of sample living with multiple conditions | 33 | 85 | | | Balance training | n/a | | 2 | | Not reported | 45 | | 4 | Nursing or residential home | No |
| **Shubert 2020 [United States]^153^** | | Pre/post design | Timed up and go (TUG); Sit to stand 30 | | Older population, with majority of sample living with multiple conditions | 42 | 75 | | | Aerobic (walking and stair climbing) exercise, balance, and resistance training | n/a | | 3 | | Not reported | 30 | | 8 | Telemedicine | No |
| **Simpson 2020 [United States]^154^** | | Parallel RCT | Frailty Index | | Inclusion criteria of T2D and obesity | 4859 | Not reported | | | Habitual physical activity (walking) | Nutrition support and behaviour change support | | 1 | | n/a | n/a | | 416 | Not reported | No |
| **Stevens- Lapsley 2022 [United States]^155^** | | Parallel RCT | Modified physical performance test; Timed up and go (TUG); Short physical performance battery; Gait speed; Handgrip | | MLTC population | n=150 Veterans (n = 135) and spouses (n = 15) | 77 | | | Resistance training | n/a | | 3 | | 8 Rep max load | 55 | | 4 | Home | Participants' spouses were also eligible for enrolment. |
| **Strasser 2018 [Austria]^156^** | | Parallel RCT | Sit to stand 30 | | Older population, with majority of sample living with multiple conditions | 117 | 82 | | | Resistance training | n/a | | 2 | | Training intensity was controlled by the colour of the therabands with the goal to perform maximally 15 repetitions per exercise. | 60 | | 76 | Nursing or residential home | No |
| **Suh 2017 [South Korea]^157^** | | Parallel RCT | Timed up and go (TUG); Gait speed | | Index condition (OA), with majority of sample living with multiple conditions | 37 | 71 | | | Resistance training | n/a | | 5 | | Borg Rating of Perceived Exertion Scale. | up to 30 | | 2 | Outpatient | No |
| **Tarazona-Santabalbina 2016 [Spain]^158^** | | Parallel RCT | Frailty phenotype (fried); Edmonton Frail Scale | | Older population, with Charlson co-morbidity index indicating multimorbidity | 100 | 80 | | | Aerobic (walking and stair climbing) training, resistance, and balance training | n/a | | 5 | | Aerobic training initially at 40% of maximum heart rate increasing progressively to 65%. Strength training initially at 25% of 1 repetition maximum to 75% | 65 | | 24 | Community | No |
| **Tosi 2021 [Brazil]^159^** | | Parallel RCT | Frailty phenotype (fried) | | Older population, with majority of sample living with multiple conditions | 43 | 84 | | | Sedentary behaviour intervention - standing balance to break sedentary time | physical activity and sedentary behaviour counselling | | 5 | | Not reported | maximum 30 | | 16 | Home | Caregivers or relatives of the participants who were vulnerable to a higher risk of falling were instructed to remain at home with the participant throughout the ex­ercise duration to offer greater support and safety |
| **Tousignant 2012 [Canada]^160^** | | Parallel RCT | Timed up and go (TUG) | | Older population, with Charlson co-morbidity index indicating multimorbidity | 152 | 80 | | | Tai chi | n/a | | 2 | | Not reported | 60 | | Not reported | Not reported | No |
| **Tsekoura 2018 [Greece]^161^** | | Parallel RCT | Gait speed; Handgrip | | Older population, with majority of sample living with multiple conditions | 54 | 73 | | | Resistance and balance training, habitual physical activity (walking) | n/a | | 2 | | Borg RPE 10-12 | 60 | | 12 | Outpatient and home | No |
| **Turunen 2020 [Finland]^162^** | | Parallel RCT | Short physical performance battery | | Older population, with majority of sample living with multiple conditions | 117 | 80 | | | Aerobic (not stated) exercise, resistance and balance training, habitual physical activity (ADLs), sedentary behaviour | Behaviour change support | | 3 | | Not reported | Not reported | | 24 | Post discharge | No |
| **Ushijima 2021 [Japan]^163^** | | Non-randomised experimental study | Frailty phenotype (fried) | | Index condition (CVD), with majority of sample living with multiple conditions | 89 patients | 75 | | | Cardiac rehabilitation | n/a | | 5 | | Based on CPET and Borg RPE scale | 40 -60 | | 12 | Outpatient rehab | No |
| **VanSwearingen 2011 [United States]^164^** | | Parallel RCT | Gait speed | | Older population, with Charlson co-morbidity index indicating multimorbidity | 50 | 77 | | | Aerobic (seated stair climbing), balance and resistance training, mobility, and transfers | n/a | | 2 | | RPE | 20-30 | | 12 | Not reported | No |
| **Verceles 2018 [United States]^165^** | | Pilot RCT | Short physical performance battery; Gait speed; Handgrip | | ICU population with majority of sample living with multiple conditions | 33 | 60 | | | Aerobic (not reported) exercise, resistance and balance training, mobility, and transfers | n/a | | 3 | | Moderate to vigorous based on the heart rate, blood pressure, oxygen saturation and subjective reporting of exertion during activities using the Modified Borg Perceived Exertion Scale, or rating of perceived  exertion | 45-60 | | 8 | ICU | No |
| **Villareal, 2006 [United States]^166^** | | Parallel RCT | Modified physical performance test | | Index condition (Obesity), with majority of sample living with multiple conditions | 27 | 70 | | | Aerobic (not stated) exercise, resistance, and balance training, stretching and flexibility | Weight management | | 3 | | Not reported | 90 | | 26 | Outpatient | No |
| **Villareal 2011 [United States]^167^** | | Parallel RCT | Modified physical performance test | | Index condition (Obesity), with majority of sample living with multiple conditions | 107 | 70 | | | Aerobic (treadmill, cycling or stair climbing) exercise and resistance training | Weight management | | 3 | | Aerobic:65% peak heart rate. Resistance:65% of their one-repetition maximum | 90 | | 52 | Not reported | No |
| **Villareal 2017 [United States]^168^** | | Parallel RCT | Modified physical performance test | | Index condition (Obesity), with majority of sample living with multiple conditions | 160 | 70 | | | Aerobic (treadmill, cycling or stair climbing) exercise resistance and balance training, stretching and flexibility | Weight management | | 3 | | n/a | n/a | | 26 | Not reported | No |
| **Wang 2022 [Canada]^169^** | | Mixed methods feasibility study | FRAIL scale | | Older population, with majority of sample living with multiple conditions | 30 | 74 | | | Resistance and balance training | Nutrition support and behaviour change support | | 2 | | Not reported | Not reported | | 8 | Telemedicine | No |
| **Williams 2022 [Australia]^170^** | | Parallel RCT | Timed up and go (TUG) | | Index condition (OA), with majority of sample living with multiple conditions | 63 | 66 | | | Aerobic (walking, cycling or arm ergometry) exercise, resistance training, stretching and flexibility | Behaviour change support | | 2 | | "Determined on an as-needs basis" | 60 | | 12 | Prehabilitation | No |
| **Winzer 2019 [Austria]^171^** | | Parallel RCT | SHARE-FI | | Older population, with majority of sample living with multiple conditions | 80 | 83 | | | Resistance training | Nutritional and social support | | 2 | | Training to muscular exhaustion | Not reported | | 12 | Home | No |
| **Young 2020 [UK]^29^** | | Mixed methods: feasibility study | Clinical Frailty Scale | | Index condition (CKD), with Charlson co-morbidity index indicating multimorbidity | 64 | 75 | | | Aerobic (cycling) exercise | n/a | | 3 | | RPE 12-14 | at least 30 | | 24 | Outpatient intradialytic | No |
| **7b. Observational studies** | | | | | | | | | | | | | | | | | | | | |
| **Abizanda 2015 [Spain]^172^** | Observational research | | Frailty phenotype (fried) | Older population, with majority of sample living with multiple conditions | | 91 | | 86 | Resistance and balance training | | | Nutritional supplementation | | 5 | Not reported |  | 12 | | Nursing or residential home | No |
| **Baldasseroni 2023 [Italy]^173^** | Observational research | | Short physical performance battery | Index condition (CVD), with Charlson co-morbidity index indicating multimorbidity | | 100 | | 81 | Cardiac rehabilitation | | | n/a | | 5 | 60-70% of peak VO2 | Each session consists  of 30 min of either biking or calisthenics on alternate  days, | 4 | | Outpatient | No |
| **Chia 2016 [Singapore]^174^** | Observational research | | Frailty phenotype (fried) | Older population, with Charlson co-morbidity index indicating multimorbidity | | 117 | | 81 | Aerobic (walking) exercise, resistance, and balance training | | | Nutritional support | | 2 | Not reported | Not reported | 2 weeks prehabilitation followed by 2-6 weeks post op rehab | | Prehabilitation and postoperative rehab | No |
| **Coleman 2012 [Ireland]^175^** | Observational research | | Clinical Frailty Scale | Older population, with majority of sample living with multiple conditions | | 32 | | 83 | Resistance and balance training, mobility, and transfers | | | n/a | | Not reported | Not reported | Not reported | 6 | | Hospital inpatient | No |
| **Kamiya 2020 [Japan]^176^** | Observational research | | Frailty Index | Index condition (HF), with majority of sample living with multiple conditions | | 1592 | | 67 | Cardiac Rehabilitation | | | n/a | | 3 to 5 | Heart rate calculated using the Karvonen formula or intensity of 12 to 13 on the Borg scale. | Not reported | 20 | | Outpatient | Multidisciplinary guidance about HF management was also provided to patients and their families |
| **Lutz 2020 [United States]^177^** | Observational research | | Modified frailty phenotype using a range of functional measures | Index condition (CVD), with majority of sample living with multiple conditions | | 243 | | 70 | Cardiac rehabilitation | | | n/a | | 2 to 3 | Not reported | Not reported | Not reported | | Outpatient | No |
| **Maddocks 2016 [UK]^28^** | Observational research | | Frailty phenotype (fried) | Index condition (COPD), with Charlson co-morbidity index indicating multimorbidity | | 816 included in analysis | | 71 | Pulmonary rehabilitation | | | n/a | | 2 supervised and one home session | 80% of predicted peak oxygen consumption based on ISWT performance. Resistance training load of 60% one-repetition maximum | 60 mins exercise, 45 minutes of education | 8 | | Outpatient | No |
| **Mareschal 2017 [Switzerland]^178^** | Observational research | | Using functional or physiological frailty criteria | Index condition (Solid organ cancer), with Charlson co-morbidity index indicating multimorbidity | | 35 | | 74 | Aerobic (not specified) exercise and resistance training | | | Nutritional and psychological support | | 2 | Not reported | 45 | 104 | | Not reported | No |
| **Matsuda 2010 [Unites States]^179^** | Observational research | | Modified frailty phenotype | MLTC population | | 72 | | 71 | Resistance and balance training, functional training, mobility, and transfers, stretching and flexibility | | | n/a | | 1 | Rating of perceived exertion, target not specified | 60 | 6 | | Home | No |
| **Mazzola 2017 [Italy]^180^** | Observational research | | A modified Frailty Index (mFI) | Index condition (Solid organ cancer), with Charlson co-morbidity index indicating multimorbidity | | 76 | | 75 | Aerobic (walking) exercise | | | Nutritional support and inspiratory muscle training | | 3 | Not reported | 30 | Not reported | | Prehabilitation | No |
| **Mockford 2014 [UK]^181^** | Observational research | | Short physical performance battery | Index condition (PAD), with majority of sample living with multiple conditions | | 51 | | 71 | Aerobic (circuit based) exercise and resistance training | | | n/a | | 3 | Low intensity | 30 | 12 | | Not reported | No |
| **Neo 2021 [Singapore]^182^** | Observational research | | Hospital frailty risk score | Index condition (Solid organ cancer), with Charlson co-morbidity index indicating multimorbidity | | 200 | | 74 | Aerobic (not specified) exercise and resistance training | | | Pharmacological care, multidisciplinary care, NMES, systems change | | 4 to 5 | not reported | 30-45 | Not reported | | Hospital inpatient | No |
| **Shiozaki 2023 [Japan]^183^** | Observational research | | Frailty phenotype (fried) | MLTC population | | 33 | | 82 | Aerobic (cycle or arm ergometer) exercise, resistance, and functional training, stretching and flexibility | | | n/a | | 2 sessions per day, 6 times per week | HRR of 40-60%  Resistance training 40%-60% of 1RM. | 60 | Average duration 26.5 ± 5.8 days | | Not reported | No |
| **Tikkanen 2015 [Finland]^184^**** | Observational research | | Frailty phenotype (fried) | Older population, with Functional co-morbidity index indicating multimorbidity | | 360 | | 82 | Resistance and balance training | | | Other comprehensive geriatric assessment informed intervention: nutrition, oral health, medical care | | 1 | Not reported | Not reported | Not reported | | Outpatient | No |
| **Valenzuela 2020 [Spain]^185^** | Observational research | | Frailty phenotype (fried) | Older population, with Charlson co-morbidity index indicating multimorbidity | | 268 | | 88 | Physical activity (sit to stands and walking) | | | n/a | | 5 | Not reported | walking 3-10 mins | For the duration of hospital stay | | Hospital inpatient | No |
| **7c. Other study designs** | | | | | | | | | | | | | | | | | | | | |
| **DeVries 2015 [Netherlands]^187^** | Intervention development paper | | EFIP | Older population, with majority of sample living with multiple conditions | | 12 | | 83 | Aerobic (not specified) exercise, resistance, balance, functional training | | | Behaviour change support | | 12 | Not reported | Not reported | 90 intake session, 30 intervention session | | Outpatient | No |
| **Gustavson 2020 [USA]^189^** | Non-randomised implementation study | | Short physical performance battery; Sit to stand 5 | Older population, with Functional co-morbidity index indicating multimorbidity | | 103 | | 78 | Resistance and balance training, functional training mobility and balance | | | n/a | | Not reported | 5 | 8 repetitions max | Not reported | | Post-acute Rehabilitation | No |
| **Jiwani 2021 [USA]^190^** | Quality improvement/ service evaluation | | Timed up and go (TUG) | Index condition (T2D), with majority of sample living with multiple conditions | | 62 | | 68 | Physical activity (walking) | | | Nutrition support | | 6 | daily | not reported | Not reported | | Outpatient and home | No |
| **Rauzi 2023a [USA]^191^ᶵ** | Quality improvement/ service evaluation | | Sit to stand 30 | MLTC population | | 21 | | 65 | Resistance and functional training and physical activity (walking) | | | Behaviour change support | | 12 | 1 to 2 | 80% 1 RM | Not reported | | Telemedicine | Spouses could enrol in the programme |

| **7d. Qualitative research** | | | | | | | | | |
| --- | --- | --- | --- | --- | --- | --- | --- | --- | --- |
| **Author [Country]** | **Study design** | **Frailty measure or functional proxy** | **MLTC measure** | **Aims** | **Sample size** | **Age** | **Summary of key findings from the authors** | **Settings** | **Carer involvement** |
| **Ahund 2020 [Sweden]^192^** | Qualitative research (interviews) | FRESH screening instrument | Older population, with Charlson co-morbidity index indicating multimorbidity | “To explore the perceptions of physical activity and exercise among frail elderly patients with a severe comorbidity burden.” | 18 | 85 | - “There is a balance between what the patients perceive as meaningful and the risk of harm, in an aging body.” - “This includes aspects related to physical activity and exercise as part of daily life, goals for physical activity and exercise and the prerequisites needed.” - “Older people relate to physical activities in daily life, particularly household- and work-related activities” | n/a | No |
| **Brighton 2020 [UK]^193^** | Qualitative research (interviews) | Short physical performance battery | Index condition (COPD), with majority of sample living with multiple conditions | “To explore the experiences, needs and preferences of people living with both COPD and frailty referred for pulmonary rehabilitation, to optimize service delivery for this group.” | 19 | 78 | - “Participants experience accumulating, multidimensional loss.” - “In striving to adapt, participants report having to negotiate the right balance between independence and support.” - “Pulmonary rehabilitation contributed to this balance: as an opportunity to actively improve their own health, with encouragement and support from others.” - “These positive experiences and outcomes motivated participants to attend, despite its physical and mental challenges.” - “Owing to fluctuating health and multimorbidity, several participants experienced multiple unpredictable disruptions. Rapport with and flexibility from service providers helped overcome these disruptions for some.” - “For others, this disruption meant they no longer felt able to engage with pulmonary rehabilitation,” | Outpatient - pulmonary rehab | Yes - participated in the interviews and PPI |
| **Bundon 2011 [Canada]^194^** | Qualitative research | Timed up and go (TUG) | Older population, with majority of sample living with multiple conditions | “To examine the exercise patterns of the frailest and most physically limited individuals. The men and women were asked about their current and previous levels of exercise, if and how they perceived their health status to have influenced their abilities to engage in exercise and what, if any, barriers, and facilitators to engaging in exercise they had encountered.” | 10 | 76 | - “Health concerns were an understandable and profound deterrent to engaging in exercise.” - “The presence of often severe health problems did not adequately explain the exercise behaviours of our participants.” - “There were three types of individuals in our study (lifelong exercisers, lifelong non-exercisers and recent adopters of exercise) made decisions about exercise that reflected underlying continuities of self.” - “The lifelong exercisers considered athleticism and the social and physical dividends of being active to be integral to their sense of identity.” - “Lifelong non-exercisers preferred cerebral, domestic or social pursuits over sports and exercise and suggested that the former were central to their sense of identity.” - “Later-life adopters of exercise revealed that their decisions to begin to engage in exercise stemmed from the desire to maintain continuities in their patterns of social engagement and their personal goals.” - “These individuals privileged social roles and identities over physical activity per se and tended to engage in exercise only when they perceived it to be instrumental to maintaining their sense of self as independent and socially engaged individuals.” | n/a | No |
| **Edbrooke 2020 [Australia]^195^ᶷ** | Qualitative research (interviews) | Clinical Frailty Scale | Index condition (Solid organ cancer), with Colinet indicating multimorbidity | “To characterise the views and experiences of participants randomised to the intervention group (home based rehab for lung cancer) to understand factors impacting on program acceptability and feasibility.” | 25 | 66 | - “The majority of participants reported program benefits, both in the physical domain (reduced sedentary time and improved strength, fitness, and function) and the mental domain (motivation to keep healthy, preventing boredom). Support to self-manage symptoms was well received and many participants reported increased confidence in managing their symptoms.” - “Exercise enablers included having expert health professional support; motivation to be stronger and better prepared for future challenges; and having an achievable and familiar program that was monitored.” - “Treatment side-effects, pain from comorbidities and the weather were exercise barriers.” - “For the majority of participants, the use of a Fitbit™ activity tracker, text message exercise reminders and an exercise diary helped to promote adherence.” - “Strategies to enable exercise adherence, such as the use of inexpensive activity monitors and automated text message exercise reminders, were well received.” | Home | Wherever possible, the participant is encouraged to have their spouse/carer/friend complete the exercise program with them |

| **7e. Ongoing studies** | | | | | | | | | | | | | |
| --- | --- | --- | --- | --- | --- | --- | --- | --- | --- | --- | --- | --- | --- |
| **Author [country]** | **Study design** | **Frailty Measure or functional proxy used** | **MLTC measure** | **Total number of participants recruited** | **Age across arms** | **Study aims relevant to the review aims** | **Additional intervention** | **Duration in weeks unless otherwise stated** | **Frequency** | **Intensity** | **Time** | **Settings** | **Were cares of relatives involved in the intervention** |
| **Hurst 2023 [UK]^199^** | Mixed methods interview and survey (ongoing study, protocol paper) | Frailty phenotype (fried) and eFI | MLTC population | sample size not provided | n/a- ongoing study | The study will involve quantitative health and lifestyle assessments and semi-structured interview to explore participants’ experiences of physical activity/exercise and their attitudes to engaging in these behaviours | n/a | n/a | n/a | n/a | n/a | Outpatient | Carers will be surveyed and interviewed |

Shared symbols (**, ᶲ, ᶳ, ᶷ, ᶺ, ᶯ, ᶹ, ᶭ, ᶵ) denote papers from linked studies. The two DeVries studies describe the development and subsequent testing of the same intervention, but in different samples and therefore have not been treated as a single study. Abbreviations: 1RM, One rep max; CAD, coronary artery disease; CFS, Clinical Frailty Scale; CIRS, Cumulative Illness Rating Scale; CKD, chronic kidney disease; COPD, Chronic Obstructive Pulmonary Disease; CVD, Cardiovascular disease; DSMS, Dutch Safety Management System; EFIP, Evaluative Frailty Index for Physical Activity; HF, heart failure, HR, heart rate; HRR, heart rate reserve; ICU, intensive care unit; MLTC, multiple long-term conditions; MPI, Multidimensional Prognostic Index; OA, osteoarthritis; OARS, The Older Americans Resources and Services; PAD, peripheral artery disease; PD, Parkinsons; RCT, randomised controlled trial; RPE, rating of perceived exertion; SOF, Study of Osteoporotic Fractures Index; STS5, sit to stand 5;T2D, Type 2 Diabetes; TUG, Timed up an go.

**Appendix 8. Markers of socioeconomic status within the included studies.**

|  | **Marker of socioeconomic status** | **Number (%) of studies** | **Domain measured and n (%) or mean ± SD** | |
| --- | --- | --- | --- | --- |
| **Pockets** | Employment | 3 (2%) | Currently working (full or part time) | *n*=99 (45%) |
|  |  |  | Not working, retired or sick leave | *n*=76 (34%) |
|  | Income | 7 (5%) | Data could not be synthesised | |
|  | Benefits | 1 (1%) | In receipt of benefits | *n*=66 (66%) |
| **Prospects** | Educational attainment | 49 (34%) | No education | *n*=228 (5%) |
|  |  |  | Primary | *n*= 986 (23%) |
|  |  |  | Secondary or higher | *n*=2707 (63%) |
|  |  |  | Mean Years of education  (*k*=8 studies) | 9.32±4.53 |
|  | Literacy | 3 (2%) | Number illiterate | *n*=32 (16%) |
| **Place** | Housing | 3 (2%) | Data could not be synthesised | |
| **Other** | Composite measure of socioeconomic status | 2 (1%) | Low | *n*=135 (33%) |
|  |  |  | Medium | *n=*50 (12%) |
|  |  |  | High | *n*=16 (4%) |

**Appendix 9. Frequency of the index conditions reported within the included studies.**


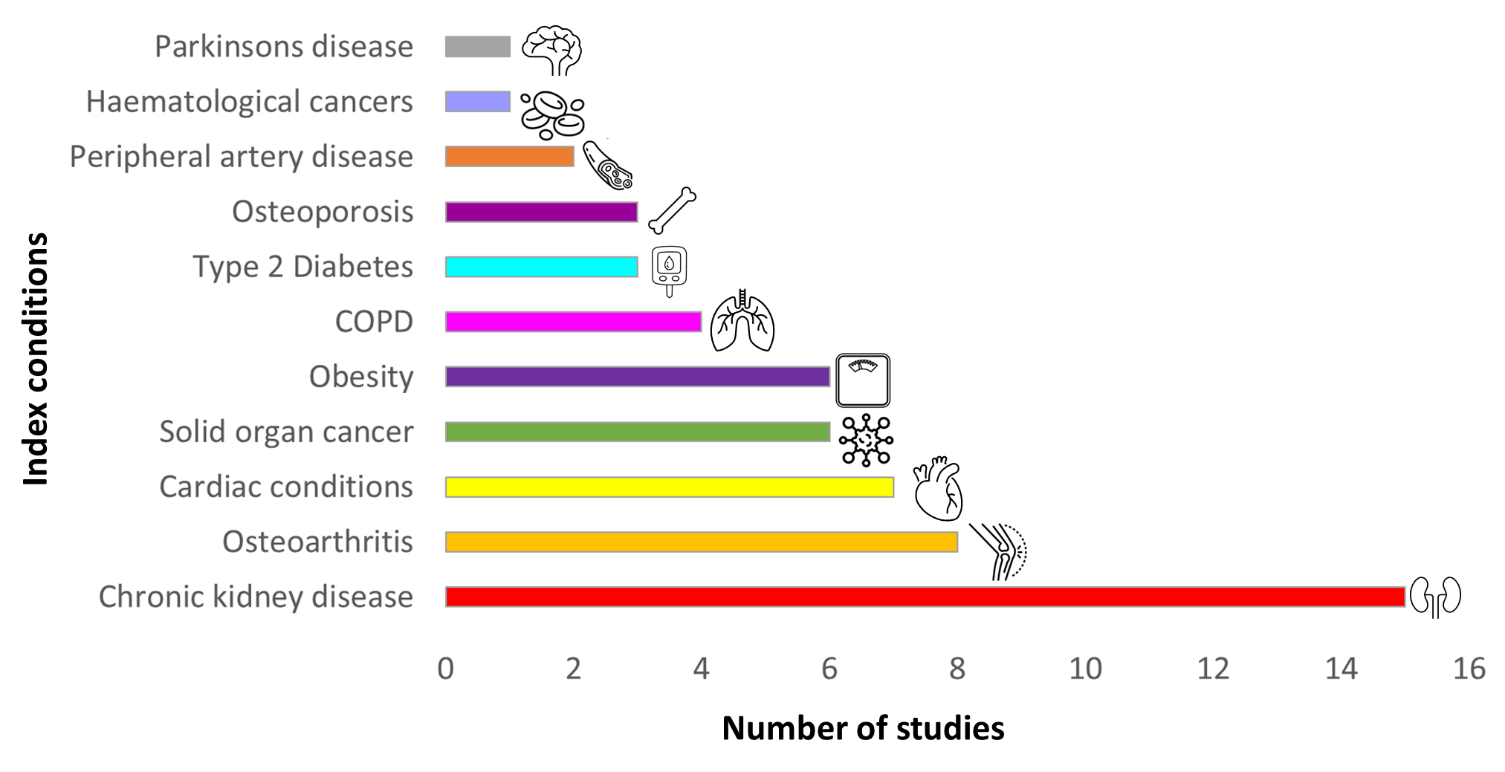


Abbreviations: COPD; Chronic Obstructive Pulmonary Disease.

**Appendix 10. Measures of co-morbidity used within the included studies, and mean values, where reported.**

| **Measures** | **Count of conditions** | **Charlson Co-morbidity Index** | **Cumulative Illness Rating Scale (CIRS)** | **Functional Co-morbidity Index** | **Colinet Co-morbidity score** | **Older Americans Resources and Services (OARS)** | **Self-administered questionnaire** |
| --- | --- | --- | --- | --- | --- | --- | --- |
| **Number of studies** | 88 (61%) | 30 (21%) | 7 (5%) | 2 (1%) | 1 (0.5%) | 1 (0.5%) | 1 (0.5%) |
| **Mean ± SD** | 3.56±1.46 | 4.41±1.98 | 8.62 ± 4.19 | 2.92±0.24 | 8.00 ± 0.61 | 5.25±2.61 | 4.45 ±2.85 |
| **Interpretation of scores** | Two or more long-term conditions is indicative of the presence of multiple long-term conditions^1^ | Medium level of co-morbidity.^247^ | Higher scores indicate greater co-morbidity.^248^ | Cumulative score indicates the number of co-morbidities.^249^ | Higher scores indicate greater co-morbidity. Measure is specific for non-small cell lung cancer.^250^ | Scores range from 0 to 14 and are derived by summing the number of comorbidities present.^193^ | Questionnaire rating the presence, severity and impact of 12 long-term conditions. Higher scores indicate greater co-morbidity and impact.^251^ |

Abbreviations: CIRS, Cumulative Illness Rating Scale; OARS, Older Americans Resources and Services.

**Appendix 11. Measures of frailty used within the included studies, and mean values (where reported) within these studies.**

| **Frailty measure** | **Number (%) of studies** | **Mean ±SD or n (%) where reported** | **Interpretation of scores** |
| --- | --- | --- | --- |
| **Fried phenotype** | 25 (17%) | 3 ± 0.7 | Frail^247^ |
| **Clinical Frailty Scale** | 6 (4%) | 5 ± 1 | Moderately frail^248^ |
| **Study of Osteoporotic Fractures Index (SOF)** | 3 (2%) | 1.71 ± 1.64 | Pre-frail^249^ |
| **Frailty Index** | 3 (2%) | 0.24 ± 0.56 | Moderately frail^250^ |
| **Evaluative Frailty Index for Physical Activity (EFIP)** | 3 (2%) | 0.34 ± 0.01 | Higher scores indicate greater levels of frailty^251^ |
| **Survey of Health, Ageing and Retirement in Europe Frailty Instrument (SHARE-FI)^252^** | 2 (1%) |  | Frail *n*=112 (64%)  Pre-frail *n*=58 (33%)  Robust *n*=5 (3%) |
| **Edmonton Frail Scale** | 2 (1%) | 8.30 ± 0.35 | Mild frailty^253^ |
| **FRAIL Scale** | 2 (1%) | 0.71 ± 0.94 | Pre-frail^254^ |
| **Tilburg Frailty Indicator** | 2 (1%) | 5.10 ± 1.05 | Frail^255^ |
| **Multidimensional Prognostic Index (MPI)^256^** | 1 (1%) | MPI- 1 *n*= 15 (14%)  MPI-2 *n*=63 (61%)  MPI-3 *n*=26 (25%) | MP1: non frail  MP2: mildly frail  MP3: severely frail |
| **Dutch Safety Management System (DSMS)** | 1 (1%) | DSMS 0 *n*=26 (8%)  DSMS 1 *n*=108 (35%)  DSMS 2 *n*=107 (35%)  DSMS 3 *n*=52 (17%)  DSMS 4 *n*=13 (4%) | Patients are at high risk of functional decline if aged 70-79 years and score > 2 or aged > 80 years and score > 1.^257^ |
| **FRESH** | 1 (1%) | 3.25 ± 0.90 | 2 or more is indicative of frailty^258^ |
| **Hospital Frailty Risk Score** | 1 (1%) |  | Low risk (<5) *n*=8 (8%)  Intermediate risk (5-15) *n*=35 (35%)  High risk (>15) *n*=57 (57%)^259^ |

Abbreviations: DSMS, Dutch Safety Management System; EFIP, Evaluative Frailty Index for Physical Activity; MPI, Multidimensional Prognostic Index, SHARE-FI, Survey of Health, Ageing and Retirement in Europe Frailty Instrument

**Appendix 12. Functional proxies for frailty used within the included studies and mean values, where reported.**

| **Functional proxy** | **Number (%) of studies** | **Mean ±SD or n (%) where reported** |
| --- | --- | --- |
| **Sit to stand 60 (reps)** | 5 (3%) | 18.07±2.67 |
| **Sit to stand 5 (seconds)** | 9 (6%) | 15.34 ± 3.47 |
| **Timed up and go (seconds)** | 41 (29%) | 13.70 ±5.32 |
| **Short Physical Performance Battery**  **(arbitrary units providing a total score)** | 22 (15%) | 6 ±2 |
| **Gait speed (m/s)** | 12 (8%) | 0.64 ±0.21 |
| **Sit to stand 30 (reps)** | 15 (10%) | 10.88 ± 2.51 |
| **Balance Performance Oriented Mobility Assessment** | 2 (1%) | 16.53 ±7.38 |
| **Modified Physical Performance Test** | 7 (5%) | 26.43 ± 7.31 |
| **Handgrip strength (kg)** | 10 (7%) | 20.31 ±6.51 |

**Appendix 13. Details of the prescription of included interventions.**

| **Frequency** | | | | | | | **Time** | | | | | | | | **Duration** | | | | | | | |
| --- | --- | --- | --- | --- | --- | --- | --- | --- | --- | --- | --- | --- | --- | --- | --- | --- | --- | --- | --- | --- | --- | --- |
| 3 (IQR 2-3) times weekly – structured exercise interventions  2 (IQR 1-2) sessions daily - habitual physical activity components, inpatient populations or nursing and residential care settings. | | | | | | | 60 mins (IQR 30-60) | | | | | | | | Range 1 week to 8 years (median 12 weeks, IQR 10-24) | | | | | | | |
| **Intensity** | | | | | | | | | | | | | | | | | | | | | | |
| **Aerobic** | | | | | | | | | **Strength** | | | | | | | | | | | **Balance** | | **Other** |
| ***HRR*** | ***HRMax*** | | ***Borg RPE*** | ***% of VO_2_ reserve*** | ***% of VO^2^ peak / proxy (e.g. ISWT)*** | ***Aerobic VT threshold*** | | ***Borg Dyspnea Scale*** | ***% Maximal force*** | ***% Maximum repetition rate*** | | ***OMNI scale*** | ***Borg CR10*** | ***Borg RPE*** | | | ***% of 10RM*** | ***% of 1RM*** | | ***Borg RPE*** | |  |
| k= 5, 4% studies | k= 7,5% studies | | k= 14, 10 % studies | k= 1, 1% study | k= 2, 3% studies | k= 2, 3% studies | | k= 1, 1% study | k= 1, 1% study | k= 1, 1% study | | k= 4, % studies | k= 1, % study | k= 13, % studies | | | k= 2, 3% studies | k= 19, 14% studies | | k= 2, 1% studies | | k= 1, 1% studies |
| 40-60%  (IQR 40-80) | 50-75%  (IQR 50-80) | | 11-14  (IQR 11-15) | 40-75% | 60-80% | Not applicable | | 4 | 30-60% | 50-70 | | 5-8  (IQR 3-10) | 5-8 | 14-16  (IQR 12-16) | | | 50-80 | 40-77  (IQR 32-80) | | 12 | | “moderate to high intensity” |
| **Decision rules for starting level** | | | | | | | | | | | | | | | | | | | | | | |
| ***Not reported k (%)*** | | | | ***Not progressive k (%)*** | | ***Started at a lower-level than target k (%)*** | | | ***Based on predefined levels k(%)*** | | | | ***Based on assessment or participant goals k (%)*** | | | | | ***Based on familiarisation sessions k (%)*** | | | | |
| 73 (52%) | | | | 1 (1%) | | 17 (12%) | | | 6 (4%) | | | | 39 (28%) | | | | | 6 (4%) | | | | |
| **Decision rules for determining progression** | | | | | | | | | | | | | | | | | | | | | | |
| ***Not reported k (%)*** | | | | ***Not progressive k (%)*** | | ***Progressive but detail not reported k (%)*** | | | ***Based on participants form/achievement of goal /RPE or OMNI level or certain number of reps /adherences to the intervention k (%)*** | | | | ***Based on achievement of a specified % level of activity at a specific time in the programme or according to a predefined progression guide k (%)*** | | | | | | | | | |
| 81 (58%) | | | | 1 (1%) | | 9 (6%) | | | 43 (31%) | | | | 39 (28%) | | | | | | | | | |
| **Tailoring of interventions** | | | | | | | | | | | | | | | | | | | | | | |
| ***Not reported k (%)*** | | ***Tailored but detail not reported k (%)*** | | ***Tailored according to conscious level k (%)*** | ***Tailored according to comorbidities k (%)*** | ***Tailored according to illness and injury k (%)*** | | ***Tailored according to physical activity levels k (%)*** | ***Tailored according to participant goals k (%)*** | | ***Tailored according to symptoms k (%)*** | | ***Tailored according to participant adherence k (%)*** | | | ***Tailored according to function and ability*** | | | ***Tailored according to stage of change k (%)*** | | ***Tailored according to frailty status n (%)*** | |
| 83 (59%) | | 6 (4%) | | 1 (1%) | 5 (4%) | 1 (1%) | | 1 (1%) | 1 (1%) | | 13 (9%) | | 2 (1%) | | | 38 (27%) | | | 1 (1%) | | 4 (3%) | |

Median (IQR) reported for intensity data unless otherwise stated. Data from two or fewer are reported directly from the paper Abbreviations: Borg CR10, Borg Category-Ratio; ISWT, Incremental Shuttle Walk Test; HRMax, Maximum Heart Rate; HRR, Heart Rate Reserve; RPE, rating of perceived exertion; RM, repetition maximum.

**Appendix 14. Ways in which informal carer support enhances physical activity engagement and adherence**

**
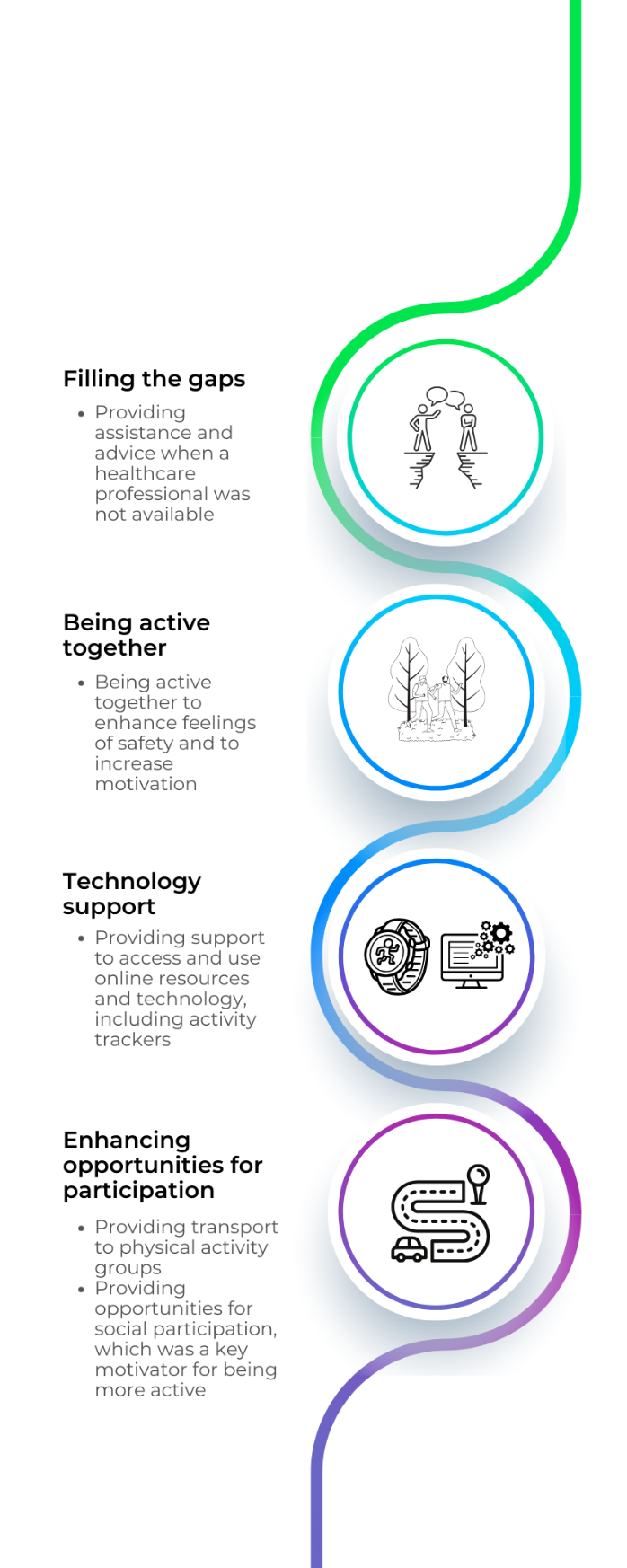
**

*Data summarised from qualitative studies included in the review^29, 162, 164, 165^.*

**Appendix 15. Reported outcomes of interventions, organised according to ICF categories.**


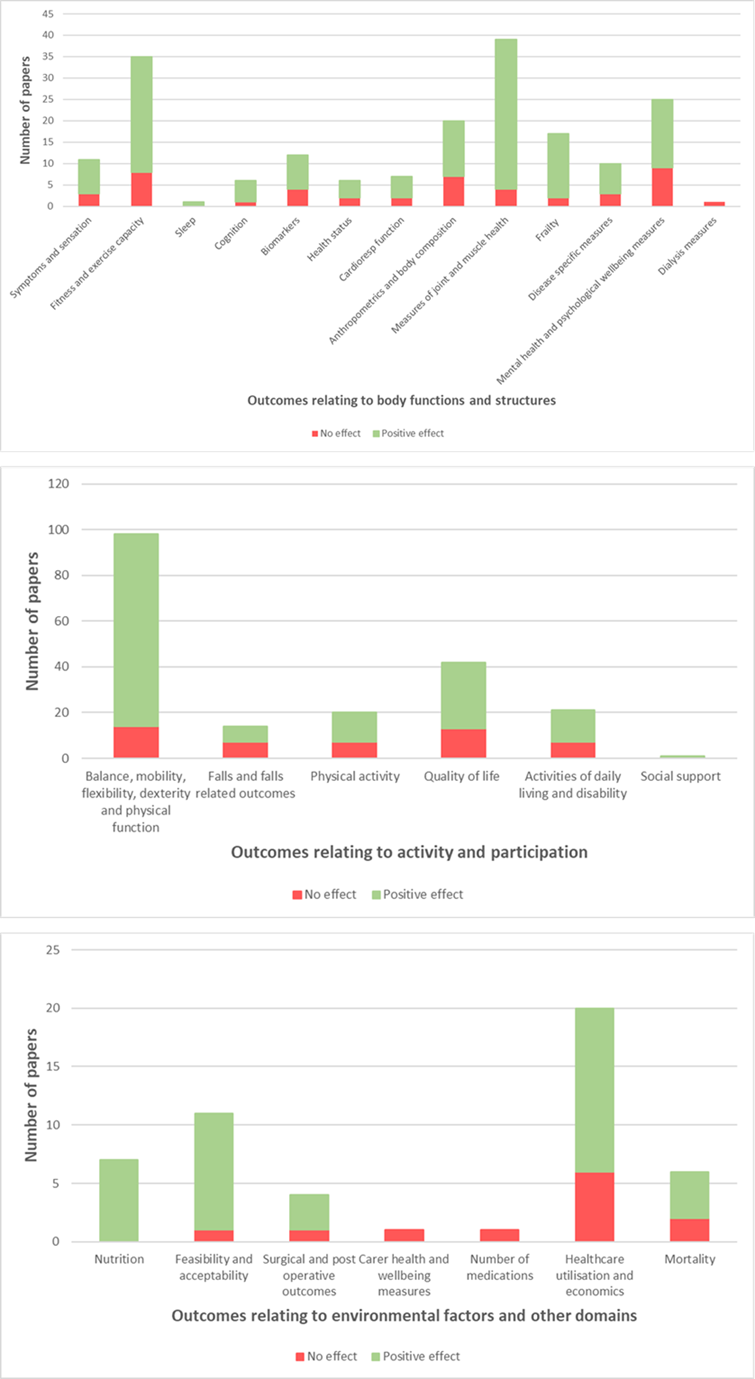


**Appendix 16. Characteristics of stakeholder group participants.**

|  | | **People living with frailty and MLTCs** | **Carers** | **Healthcare professionals** |
| --- | --- | --- | --- | --- |
|  | | *N*=7 | *N*=6 | *N*=8 |
| **Age** (years) | | 70 (51-76) | 59 (52-68) | 34 (32-43) |
| **Sex** *n* (%) | Female | 6 (86%) | 4 (67%) | 4 (50%) |
|  | Male | 1 (14%) | 2 (33%) | 4 (50%) |
| **Ethnicity** *n* (%) | White British | 0 (0%) | 1 (17%) | 8 (100%) |
|  | Asian or Asian British | 4 (57%) | 4 (67%) | 0 (0%) |
|  | Black or Black British | 3 (43%) | 1 (17%) | 0 (0%) |
| **Educational level**  *n* (%) | Secondary | 5 (71%) | 3 (50%) |  |
|  | Undergraduate | 1 (17%) | 1 (17%) |  |
|  | Other | 0 (0%) | 1 (17%) |  |
|  | Information not provided | 1 (14%) | 1 (17%) |  |
| **Professional background** *n* (%) | Academic –sarcopenia and LTCs |  |  | 2 (25%) |
|  | Exercise professional or physiotherapist |  |  | 2 (25%) |
|  | Geriatrician |  |  | 1 (12%) |
|  | Physician |  |  | 1 (12%) |
|  | General Practitioner |  |  | 1 (12%) |
|  | Behavioural Scientist |  |  | 1 (12%) |
| **Professional experience** (years) | |  |  | 10 (5-19) |
| **Clinical frailty Scale Score** | | 5 (4-6) | 0 (0-4) |  |
| **Number of long-term conditions** | | 3 (2-3) | 2 (2-4) |  |
| **Relationship to care recipient** | Adult child |  | 2 (33%) |  |
|  | Parent |  | 2 (33%) |  |
|  | Spouse |  | 1 (17%) |  |
|  | Multiple carer |  | 1 (17%) |  |
| **Average time spent caring per week** (hours) | 90+ |  | 5 (83%) |  |
|  | 50-89 |  | 0 (0%) |  |
|  | 20-49 |  | 0 (0%) |  |
|  | 1-19 |  | 1 (17%) |  |
| **Caring duration** (years) | 15+ |  | 3 (50%) |  |
|  | 10-14 |  | 0 (0%) |  |
|  | 5-9 |  | 1 (17%) |  |
|  | 1-4 |  | 2 (33%) |  |
|  | Less than 1 year |  | 0 (0%) |  |
| **Clinical frailty Scale Score of the care recipient** |  |  | 7 (6-7) |  |
| **Number of long-term conditions of the care recipient** |  |  | 2 (1-6) |  |

*Median and IQR are reported for continuous variables.LTCs, long term conditions; MLTCs, multiple long term conditions.*

**Appendix 17. Results of the consultation process and joint display combining key findings from the review with key themes from stakeholder engagement.**

Consensus across areas for future research was consistent, with different stakeholder groups tending to emphasise different areas which they had all identified as important. All stakeholders shared concerns that existing research may not accurately represent individuals with lower socioeconomic status, diverse ethnic backgrounds, mental health-related MLTCs, and severe frailty. They also unanimously prioritised mental health, QoL and life participation as crucial outcomes for future studies and emphasised the importance of measuring outcomes after intervention cessation to evaluate sustainability and longer-term effects.

All participants also all emphasised the need for further evaluation of sedentary behaviour and light physical activity interventions as foundational components preceding traditional moderate-to-vigorous activity structured exercise. These lighter activities may extend outside ‘conventional’ physical activity, offering inclusivity for those unable to meet recommended guidelines, and reinforcing the message that 'all movement counts.' Light physical activity was seen as more enjoyable, sustainable, and culturally relevant, fostering motivation and self-efficacy for more intense activities in the future.

Finally, addressing carer health emerged as a significant gap identified by all stakeholders. All groups agreed on the importance of involving carers in intervention development and flexibly negotiating and agreeing on the level of support from carers during the intervention.

Healthcare professionals (HCPs) expanded further on several of these points. Regarding research design they emphasised the importance of improved reporting of socioeconomic status and ethnicity with future research. They also acknowledged the challenges in accurately measuring QoL and advocated the development of a specific, validated MLTC QoL measure, or suite of measures. In contrast the carer group emphasised the significant unmet support needs of the carer group, including challenges meeting their physical health and mental health needs due to caring responsibilities.

Regarding interventions, people living with frailty and MLTCs linked inclusion to the types of interventions evaluated and felt some of the interventions studied were not accessible, sustainable, or enjoyable for these under-represented groups. In contrast, the HCP group recognised a crucial need to understand how to engage people living with frailty and MLTCs in resistance training, and the reasons for sedentary behaviour in this population. They also identified tailoring for combinations of different MLTCs and for frailty level as an important area for future research.

| **Research question** | **Key findings from the review** | **Key stakeholder perspectives from the consultation process** | | |
| --- | --- | --- | --- | --- |
|  |  | **Healthcare professional and researchers** | **People living with frailty and MLTCs** | **Carers** |
| Characteristics of physical activity, exercise and sedentary behaviour interventions | - Eighty-three studies (60%) evaluated a structured exercise intervention. - *k*=2 (1%) habitual physical activity and *k*=1 (0.7%) sedentary behaviour as stand-alone interventions. - Habitual physical activity and sedentary behaviour interventions are also under-represented in combined interventions. - Habitual physical activity interventions focus on walking but many people with frailty and MLTC see activity as everyday ‘work’ and activities of daily living. - The value of resistance training does not appear to be well understood or explored within current qualitative research. - No qualitative studies exploring sedentary behaviour were identified. - Home-based interventions have most often been studied and appear to be viewed as less burdensome and more acceptable. - Tailoring in relation to individual abilities and symptoms was most often reported, and tailoring for multiple conditions and frailty status was less common. | Interventions focusing on the role of light/ habitual physical activities represent important options for groups of people unable to meet guidelines relating to moderate and vigorous physical activity or structured exercise | | |
|  |  | Lighter intensity activities are helpful ‘building blocks’ to other activities and enable people to build spatial awareness, proprioception and balance which can help them to be successful as they progress to MVPA. | | |
|  |  | Typical lighter physical activity such as walking should not be prioritised at the expense of other activities such as dance and using ADLS as activity. | | |
|  |  | Dance may be culturally relevant and support an increased sense of identity | ADLS as lighter intensity activities were identified as easier to sustain | |
|  |  | Resistance training was identified as crucial by HCPs but challenging to implement and sustain. More research in this area would be beneficial. |  |  |
|  |  | Qualitative research exploring why this population are sedentary would be helpful for intervention development – for example low motivation requires a very different intervention to increased sedentary behaviour due to ill-health. |  |  |
| Inclusion of carers and relatives | - Carers were rarely included in the identified studies. - Where they were involved, this was at the behest of the person with frailty and MLTC and focused upon improving their engagement and adherence. - The characteristics of the carers involved in these studies was difficult to determine. - Outcomes relating to carer health have been understudied and do not appear to show positive outcomes. | Further research focusing on carer health and carer wellbeing was supported by all stakeholder groups. | | |
|  |  | All groups agreed that whether carers are involved in an intervention for those they care for should be determined on an individual basis, considering the wishes of both parties. The involvement of carers should not be assumed. | | |
|  |  | All groups also agreed that caring relationships, attachment styles and family dynamics vary. Time for both groups to engage in joint activities is also challenging. These factors may act as both barriers and facilitators to engagement and adherence. | | |
|  |  |  |  | Carers reported experiencing significant inequality, lack of support and subsequently challenges with their mental health due to cultural expectations to undertake a caring role, stress, inability to access services to support their health needs, guilt, anxiety, isolation, loss of confidence, exhaustion and lack of sleep. |
|  |  |  |  | Carers wanted an intervention to support their mental health and wellbeing |
|  |  |  |  | Carers reported that professional support with exercise and guidance with moving and handling to prevent injury when supporting physical activity programmes was important. |
| Engagement and adherence | - Adherence with the intervention was 81%. - Goal setting, monitoring, feedback, peer, professional and carer/ family support was important to ongoing engagement, as was previous experiences of physical activity and exercise. - Qualitative data revealed that the decision to participate in physical activity was a process of weighing up the potential benefits with anticipated risks and concerns about safety. | Selecting the most appropriate form and level of activity for the individual should a shared process in which key people are all involved. This process can be used to communicate *why* an activity is important, focusing on the priorities of the individual rather than ‘generic’ benefits and avoidance of future health risk, which may not be compelling to this population. | | |
|  |  | Support to help people form habits could also be relevant to sustaining behaviour change in the longer term and may be especially relevant to this population where health, symptom and treatment burden can interfere with maintaining behaviours. | | |
|  |  | Pain, poor mental health and the loss of safe community venues supporting activity were recognised barriers to activity across all groups. | | |
|  |  | Peer and professional support were identified as important |  | Peer and professional support were identified as important |
|  |  |  | People with MLTCs and frailty underlined the importance of accessible, low cost, simple and fun physical activity options.  Tracking activity was also felt to be important, and many people were already tracking their activity, those who weren’t were interested in doing so. |  |
| Outcomes | - Outcomes focused primary on body functions and structures and to a lesser extent activity and participation. - Studies appear to show that interventions are safe and effective in this population, but a systematic review is required to be assured of this. | Outcomes should be measured beyond the cessation of the intervention to determine longer term effects and maintenance of behaviours. | | |
|  |  | HCP professionals identified a need for research focused on improving quality of life.  HCPs understood the greater focus on more objective measures and measures of healthcare utilisation at the expense of QoL measures in the existing literature. QoL of life was felt to be challenging to measure in the context of MLTCs.    Measures for single LTCs may not   - be validated for MLTCs - capture what is important in this population.   A specific MLTC measure which captures:   - QoL elements which are specific to specific conditions within the MLTC cluster - how perceptions of QoL change across the life course   may be required. |  | Mental health outcomes were of primary importance |
| Other | - Only eight (5%) of studies were conducted in LMIC. - Participants were older, with a mean age of 73 ± 12 years. - Where data on ethnicity and socioeconomic status were reported, White affluent populations were most often included. - MLTCs relating to physical health conditions seem to have been prioritised over mental health conditions. | The lack of inclusion of people with mental health MLTCs was identified as a significant gap across all groups. This gap was felt to be significant due to the bidirectional relationships between physical and mental health, and the impact of mental health on motivation and engagement in movement behaviour interventions. | | |
|  |  | Better reporting of ethnicity and socioeconomic status was identified as important by HCPs to determine if the participants involved where representative of the population  The proportion of participants with English as a first language, and who required carer support was also felt to be important to measure. |  |  |
|  |  | HCPs highlighted a need to focus research on seldom heard and under privileged communities where need is greatest. | People with frailty and MLTC highlighted a need to focus research on seldom heard and under privileged communities where need is greatest. People with MLTCs and frailty spoke about challenges with trusting researchers following previous experiences of poor healthcare. They also discussed the need for accessible physical activity interventions – some options (e.g., yoga, hydrotherapy and Pilates) they had not heard of before and may not be readily available in their communities |  |
|  |  | Participants with more severe frailty were also noted to be challenging to engage in research especially relating to physical activity. Recruitment and retention approaches that have been successfully used in the field of palliative care may be helpful within the field of MLTCs and frailty. |  |  |

Perspectives shared by all stakeholders cut across all columns, whilst views specific to a particular stakeholder group are presented separately. Abbreviations: HCP, health care professionals; LTCs, long term conditions; MLTCs, multiple long-term conditions.
